# Clinical efficacy evaluation and potential mechanism prediction on Pudilan Xiaoyan oral liquid in treatment of mumps in children based on meta-analysis, network pharmacology, and molecular docking

**DOI:** 10.3389/fphar.2022.956219

**Published:** 2022-09-23

**Authors:** Yi Liu, Xin Cui, Junyu Xi, Yanming Xie

**Affiliations:** Institute of Basic Research In Clinical Medicine, China Academy of Chinese Medical Sciences, Beijing, China

**Keywords:** meta-analysis, network pharmacology, molecular docking, mumps, Pudilan Xiaoyan oral liquid, traditional Chinese medicine, randomized controlled trials

## Abstract

**Background:** Mumps is caused by the mumps virus and is characterized by pain and parotid gland swelling. Although its incidence has declined due to vaccines, outbreaks still occur among children. In addition, it can lead to severe complications, so it has a certain perniciousness. Pudilan Xiaoyan oral liquid (PDL), a Chinese patent medicine, commonly treats children with mumps. However, its safety, efficacy, and specific mechanisms lack relevant evaluation and analysis. Therefore, we did a meta-analysis of the randomized controlled trials combined with a network pharmacology analysis to assess the efficacy and safety of PDL in relieving symptoms of mumps in children and investigate its pharmacological mechanisms.

**Methods:** This study systematically searched the China National Knowledge Infrastructure (CNKI), WanFang Data Knowledge Service Platform, VIP Database, Sinomed, Chinese Medical Journal Full-text Database, PubMed, Embase, Cochrane Library, Web of Science, and Google Scholar for the published randomized controlled trials (date up to 3 March 2022; studies in both English and Chinese) comparing PDL and antiviral drug combination treatment to standalone antiviral drug treatment. The primary outcomes in this study were the effective rate and duration of five characteristic symptoms of children’s mumps. We assessed the pooled data by using a fix-effect or random-effect model. We illustrated an odds ratio (OR) or standardized mean difference (SMD) with a 95% confidence interval (CI) using the Stata 15 software. In network pharmacology, active components of PDL were collected from the traditional Chinese medicine system pharmacology technology platform and the CNKI studies, while mumps’ targets were collected from databases of the Genecards and Online Mendelian Inheritance in Man (OMIM), and then we constructed a “drug-component-target” network and a protein–protein interaction network using Cytoscape 3.9.0 for screening the core components and targets. Next, we ran Gene Ontology (GO) and Kyoto Encyclopedia of Genes and Genomes (KEGG) analysis of intersection targets of PDL and mumps. Finally, molecular docking was performed between core components and targets.

**Results:** Of 70 identified studies, 12 were eligible and included in our analysis (N = 1,307 participants). Compared with the antiviral drug treatments, combination treatment using PDL and antiviral drugs provided higher effective rates (OR = 5.94), shorter symptom durations for fever (SMD = −1.05), headache (SMD = −0.69), parotid gland swelling (SMD = −1.30), parotid gland pain (SMD = −2.53), and loss of appetite (SMD = −0.56) with fewer reported side effects. Of the 113 active components of PDL and 57 mumps’ targets, 11 core components like quercetin, isoetin, and seven core targets such as albumin (ALB) and interleukin-6 were obtained. Moreover, the potential pathways identified included cytokine–cytokine receptor interaction and T helper cell 17 (Th17 cell) differentiation. Molecular docking results revealed that most core components and targets could form stable structures. The core components, including isoetin, quercetin, and luteolin, and core targets involving heat shock protein HSP 90-alpha (HSP90AA1), estrogen receptor (ESR1), and ALB showed the best affinities.

**Conclusion:** The combined use of PDL and antiviral drugs could effectively improve the efficacy of mumps among children and rapidly alleviate mumps-related symptoms. This efficacy may be associated with the anti-inflammatory and antiviral mechanisms by which PDL acts using multiple components, multiple targets, and multiple pathways. However, these results should be confirmed by further studies.

## 1 Introduction

Mumps is a common infectious disease among children caused by the mumps virus (MuV) (Su et al., 2020), whose contagious characteristics involve swelling and parotid gland pain (Hviid et al., 2008). Although its incidence has decreased with MuV vaccination, most MuV transmission chains have not been effectively interrupted (Cui et al., 2017), and its outbreaks are frequently reported. For instance, Dyer (2017) observed that mumps in the United States increased nearly fivefold in 2017 than in 2015; Opstelten et al. (2012) found recurring mumps outbreaks in Dutch since 2009, especially among students. Mumps is also high in mainland China, with an annual incidence of 24 cases per 100,000 people (Peng et al., 2021). In some countries having low coverage of MuV vaccination, the situation is even more challenging (Doshi et al., 2017). Mumps can also cause serious complications such as orchitis, arthritis, meningitis, pancreatitis, and encephalitis, especially in children (Ohfuji et al., 2021). Therefore, seeking a safe and effective drug to treat mumps is crucial.

In China, traditional Chinese medicine (TCM), with a unique theory and a long history, provides safe and effective complementary and alternative treatments for many diseases, including mumps. Some studies (Qi et al., 2020; Sun X. et al., 2021; Lyv, 2021) have shown that Chinese patent medicines, oral or topical Chinese medicines, can effectively ease mumps-related symptoms and reduce cytokine levels or serum amylase and serum amyloid A levels. Pudilan Xiaoyan oral liquid (PDL) is a traditional Chinese patent medicine preparation from TCM theory and is mainly composed of four different herbs, including Pugongying (Pharmaceutical Latin: *Herba Taraxaci;* English name: dandelion), Kudiding (Pharmaceutical Latin: *Herba Corydalis Bungeanae;* English name: *Corydalis bungeana*), Banlangen (Pharmaceutical Latin: *Radix Isatidis*; English name: woad root), and Huangqin (Pharmaceutical Latin: *Radix Scutellariae*; English name: baical skullcap root). All four herbs have the effects of removing heat and toxic materials, cooling blood, and reducing swelling. Thus, PDL is widely used in treating various kinds of inflammatory diseases. Animal experiments (Deng et al., 2020; Shi et al., 2021) have observed that it has a therapeutic effect on *Streptococcus pneumoniae* infection in mice and has an inhibitory effect on the severe acute respiratory syndrome coronavirus 2 (SARS-CoV-2). A systematic review (Bian et al., 2017) reported that PDL could treat suppurative tonsillitis in children. Some clinical studies on PDL (Wu and Ren, 2021; Zhou and Hou, 2021; Zou and Liu, 2021) have revealed therapeutic effects on suppurative otitis media, hand-foot-mouth disease, and pharyngitis among children.

PDL is also widely used in treating mumps clinically. Nevertheless, the studies on PDL treatment of mumps are limited to case reports, experience summaries, and clinical observations. Not only the level of evidence is low, but also there are few studies deciphering the mechanisms. Thus, its specific safety, efficacy, and mechanisms still need further evaluation. A meta-analysis, as a summary and objective and quantitative analysis of previous results, provides the best evidence in evidence-based medicine (Lee, 2018), while a meta-analysis of randomized controlled trials (RCT) can evaluate both the safety and efficacy of drugs in treating diseases (Palmowski and Nielsen, 2020). In comparison, network pharmacology can analyze the molecular relationships between drugs and diseases and elucidate the systemic pharmacological mechanisms from system levels and overall biological networks to guide the designing of new drugs, clinical diagnosis, and treatment (World Federation of Chinese Medicine Societies, 2021). Therefore, this article systematically screened out studies that met the quality standards to accurately evaluate the efficacy and safety of PDL in treating mumps by using meta-analysis and network pharmacology. Furthermore, we conducted a predictive analysis of the potential mechanisms of PDL in the treatment of mumps to provide references to clinical applications and future research.

## 2 Methods

### 2.1 Retrieval strategy

The current meta-analysis was registered at the International Prospective Register of Preferred Reporting Items (PROSPERO) (Number: CRD42022327802) and followed the Preferred Reporting Items for Systematic Reviews and Meta-Analyses (PRISMA) statement.

The relevant studies were collected from the beginning of each database to March. 3, 2022. The databases we retrieved included the China National Knowledge Infrastructure (CNKI), WanFang Data Knowledge Service Platform (WanFang), VIP information resource integration service platform (VIP), Sinommed, Chinese Medical Journal Full-text Database (Yiigle), PubMed, Cochrane Library, Embase, Web of Science, and Google Scholar. The language of retrieval was limited to Chinese and English. Moreover, an advanced search was applied in every database, combined with the subject heading words and logic operator. The specific search strategies are listed in Supplementary Table S1.

### 2.2 Selection criteria

#### 2.2.1 Inclusion Criteria

All the studies retrieved were regarded as eligible for inclusion if they met the following criteria: if the patients were children having mumps (diagnosed based on Zhu Futang Practical Pediatrics (Hu and Jiang, 2002) or the Pediatric Treatment Guidelines (Wu, 2012)); if patients were treated using PDL or PDL combined with antiviral drugs (with or without basic symptomatic treatment) within the intervention group; if the patients were treated without PDL or another TCM formulae in the control group and provided the same basic symptomatic treatment as the intervention group; if the studies reported changes in effective rate, symptoms, and indicators of inflammation and infection; if the studies included were RCTs.

### 2.2.2 Exclusion criteria

Exclusion criteria involved animal experiments, observational and retrospective studies, reviews, and case reports; mumps in adults; studies not assessing PDL, antiviral treatment, and primary symptomatic treatment; studies assessing other TCM treatments. If there was a study with duplicate data, the one with more essential data was selected.

### 2.3 Data extraction

Two independent investigators filtered the studies based on the inclusion criteria. First, they imported studies by retrieving subject headings into Endnote software to check for duplicates. After that, they reviewed the titles and abstracts to judge if the studies followed the inclusion criteria. Finally, they performed a full-text assessment to determine whether the studies could be included. In case of disagreement, third-party personnel would participate in the discussion.

This study used Endnote and Microsoft Excel software to create a data extraction table. The extracted data included the first authors’ names, year of publication, the total number of participants, age, gender, literature quality, interventions (including dosage and duration of treatment), outcome indicators, etc. Two independent investigators evaluated the methodological quality of the articles and consulted with third-party personnel when opinions differed.

### 2.4 Quality assessment

The quality of the included studies was assessed through the Cochrane tool of risk of bias assessment (Higgins et al., 2022a), primarily in terms of random sequence generation, allocation concealment, blinding of participants and personnel, blinding of outcome assessment, incomplete outcome, and selective reporting, and other biases were evaluated based on seven aspects. The included studies were finally scored as “low risk,” “high risk,” and “unclear."

### 2.5 Components and targets in PDL

This study utilized the traditional Chinese medicine system pharmacology technology platform (TCMSP, http://tcmspw.com/tcmsp.php) (Ru et al., 2014) and the CNKI reviews to retrieve the components of PDL and based on oral bioavailability (OB) ≥ 30% and drug-likeness (DL) ≥ 0.18 to filter for the components that are not included in the TCMSP database. The SwissADME (http://www.swissadme.ch/) (Daina et al., 2017) database was used for screening using filter conditions: OB is “high,” and DL has more than three “yes” items. Then, the Universal Protein (Uniprot, http://uniprot.org/) (The UniProt Consortium, 2018) database was utilized to match relevant targets for components available in the TCMSP or the SwissTargetPrediction (http://www.swisstargetprediction.ch/) (Daina et al., 2019) to predict targets for the components not available in the TCMSP.

### 2.6 Mumps-related targets

This study searched the Gene Expression Omnibus (GEO, https://www.ncbi.nlm.nih.gov/) (Clough and Barrett, 2016), Genecards (https://www.genecards.org/) (Safran et al., 2021), Online Mendelian Inheritance in Man (OMIM, https://omim.org/#) (Amberger et al., 2015), and the Therapeutic Target Database (TTD, http://db.idrblab.net/ttd/) (Zhou et al., 2022) for “mumps,” “mumps virus,” and “parotitis” to retrieve the mumps-related targets, merged, and deduplicated the mumps-related targets secured from the four databases.

### 2.7 Construction of “drug-component-target” network

Excel software was used to obtain the intersection of the relevant targets for each active component in PDL and the mumps-related targets. Then, they took the disease, drugs, components, and related targets as nodes and their mutual relationship as edges to construct a network by using Cytoscape 3.9.0. We underwent a topological analysis to retrieve the core components.

### 2.8 Construction of protein–protein interaction network

The PPI network of the intersection targets was acquired from the String (https://string-db.org) (Szklarczyk et al., 2021) database by selecting the species as *Homo sapiens*, the minimum required interaction score as 0.40, and the core PPI targets were obtained through the MCODE plugin for clustering the PPI network.

### 2.9 Enrichment analysis

The intersection targets were imported into the Metascape (https://metascape.org/) (Zhou et al., 2019) platform for undergoing Gene Ontology (GO) enrichment analysis of biological processes, molecular functions, cellular components, and the Kyoto Encyclopedia of Genes and Genomes (KEGG) pathway enrichment analysis. Then, we used the ImageGP platform (http://www.ehbio.com/ImageGP/) (Chen et al., 2022) to draw the bubble plots for GO and KEGG analysis.

### 2.10 Molecular docking

The 3D structures of the previously obtained core components and targets, MuV, ribavirin, and ganciclovir (as controls), were acquired from Pubchem (https://pubchem.ncbi.nlm.nih.gov) (Kim et al., 2019) and PDB (http://www.rcsb.org/) (Berman et al., 2000) databases. Then, we used Autodock Vina for molecular docking and selected the best four combinations of docking using Pymol for visualization.

### 2.11 Statistical analysis

The meta-analysis used STATA version 15.0 software to perform the statistical analysis. Standardized mean difference (SMD) and the odds ratio (OR) were selected as effect quantity, and the interval estimation was expressed with a 95% confidence interval (CI). When the homogeneity among the studies was low (*p* ≥ 0.1, I^2^ ≤ 50%), a fixed-effect model would be selected for analysis. In contrast, when the heterogeneity among the studies was significant, we analyzed the reasons first. If the causes were clinical factors or research methods, we performed a subgroup analysis depending on intervention differences and sensitivity analysis. If the heterogeneity were still significant after the study (*p* < 0.1, I^2^ > 50%), a random-effect model would be selected for analysis (Deeks et al., 2022b). Simultaneously, when more than 10 studies were included in the outcome indicators, a funnel plot and Begg’s and Egger’s tests were used to determine whether there was a publication bias. *p*-value < 0.1 was considered a significant publication bias. In network pharmacology, the topological analysis of the “drug-component-target” network was performed with the network analyzer plugin of Cytoscape, and the core components were screened out through their node degrees. In the PPI network analysis, we utilized the MCODE plugin of Cytoscape and set the degree cut-off = 2, node score cut-off = 0.2, K-core = 2, and max. depth = 100 to find clusters in the PPI network, and we also screened out the core targets by their node degree. The GO function enrichment analysis and KEGG pathway enrichment analysis were undergone by setting the *p*-value cut-off to 0.01, min enrichment to 1.5, min overlap to 3, and the Q-value equal to or less than 0.01 was considered statistically significant. As for molecular docking, we set the energy range = 5, exhaustiveness = 400, and the number of models = 20 to obtain the binding energy combinations.

## 3 Results

### 3.1 Results of included studies

**FIGURE 1:**
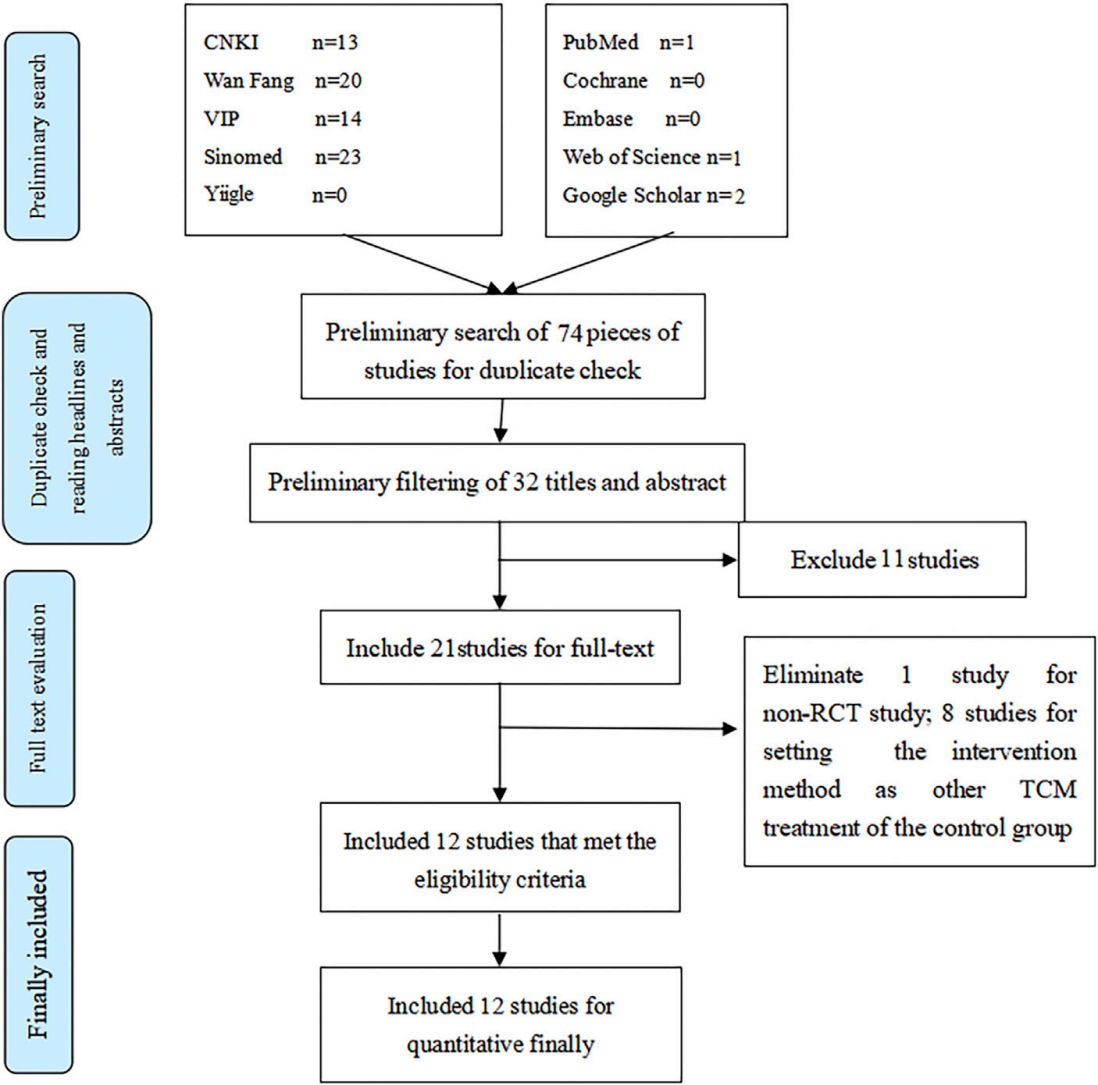
Flow diagram of the study selection.

Twelve studies were included in our analysis from 74 identified studies, most in Chinese. The flowchart is shown in Figure 1. The 12 trials were published between 2010 and 2020 with 1,307 participants, of which 659 were in the test group, while 648 were in the control group. More details can be seen in Table 1.

**TABLE 1:** Characteristics of the included studies.

No.	Information of studies	Sample size			Gender male/female		Average age/years		Interventions		Treatment duration/d	Outcomes
		T	C	Total	T	C	T	C	T	C		
1	Chang (2020)	43	43	86	24/19	23/20	10.5 ± 1.4	10.3 ± 1.1	PDL 10ml, tid + ganciclovir 5 mg/kg, bid	Ganciclovir, 5 mg/kg, bid	5	①②④⑤
2	Gao (2019)	58	58	116	32/26	30/28	2.00 ± 0.89	2.00 ± 0.95	PDL 0.8–1.0 ml/(kg·d) + ribavirin 10–15 mg/(kg·d) + CT	Ribavirin 10–15 mg/(kg·d) + CT	5–7	①②③④
3	Jia (2012)	40	40	80	43/37		8		PDL 7.5–10 ml, tid + ribavirin 10–15 mg/(kg·d) + CT	Ribavirin 10–15 mg/(kg·d) + CT	7	①
4	Jiang (2013)	51	50	101	27/23	22/28	8.0 ± 1.4	7.5 ± 1.8	PDL 5–10 ml, bid + ribavirin 100 mg, bid	Ribavirin 100 mg, bid	7	①②③④⑤⑥
5	Lan and Chen(2012)	68	68	136	76/60		4–15		PDL 2.5–10 ml, tid + ribavirin 10–15 mg/(kg·d)	Ribavirin 10–15 mg/(kg·d)	7	①②③④⑦
6	Li(2015)	145	144	289	86/59	77/67	3∼12	4∼15	PDL 1.0 ml/(kg·d), tid + ribavirin 10 ml/(kg·d)	Ribavirin 10 mL/(kg·d)	5	①
7	Liang (2018)	41	41	82	23/18	24/17	8.72 ± 1.43	8.86 ± 1.58	PDL 10 ml, tid + ganciclovir 10 mg/(kg·d)	Ganciclovir 10 mg/(kg·d)	5	①②④⑤
8	Ning (2019)	37	37	74	22/15	20/17	7.16 ± 1.71	7.30 ± 1.80	PDL 3–10 ml, tid + ganciclovir 10mg/(kg·d)	Ganciclovir 10 mg/(kg·d) + CT	5	①②④⑤⑧
9	Wang and Liu(2010)	45	45	90	48/42		7.58		PDL 2.5–1 ml, tid + ribavirin 10–15 mg/(kg·d) + CT	Ribavirin 10–15 mg/(kg·d) + CT	5	②③④⑨
10	Zhang and Ding(2013)	55	54	109	31/24	24/25	6.30 ± 1.82	6.12 ± 1.7	PDL 2.5–1 ml, tid + ribavirin 10–15 mg/(kg·d) + CT	Ribavirin 10–15 mg/(kg·d) + CT	7	①②③④⑥
11	Zhou et al. (2014)	38	30	68	42/26		8.4		PDL 0.8–1.0 ml/(kg·d) + acyclovir 7.5 mg/kg, bid + CT	Ribavirin 10–15 mg/(kg·d) + CT	5	①②③④
12	Zhu (2019)	38	38	76	21/17	20/18	8.02 ± 1.59	8.19 ± 1.58	PDL 10 ml, tid + ganciclovir 10 mg/(kg·d)	Ganciclovir 10 mg/(kg·d)	5	①②④⑤⑩

Note: T: test group; C: control group; CT: conventional treatment. The baseline is balanced. ① Effective rate; ② duration of fever; ③ duration of headache; ④ duration of parotid gland swelling; ⑤ duration of parotid gland pain; ⑥ duration of loss of appetite; ⑦ duration of sore throat; ⑧ levels of C-reactive protein (CRP), interleukin 6 (IL6), and tumor necrosis factor-alpha (TNF-α) in peripheral venous blood; ⑨ duration of vomiting; ⑩ length of hospital stay and cost of medical care.

### 3.2 Bias assessment

**FIGURE 2:**
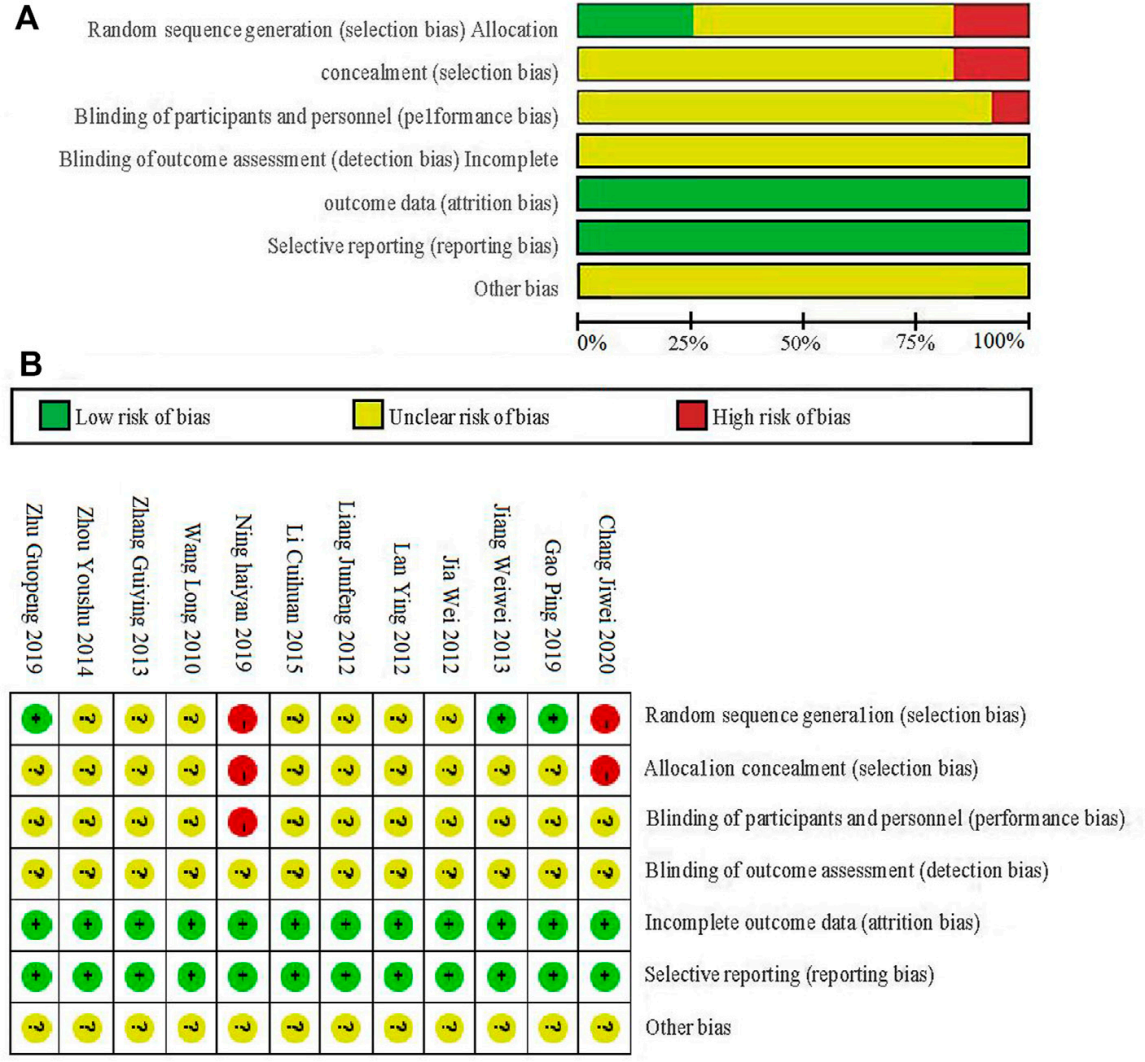
Overall risk **(A)** and detailed risk **(B)** of bias in the included studies.

We assessed the quality of included studies based on the Cochrane tool for risk of bias assessment. The included studies were RCTs, three of which reported specific randomization methods, including random number tables and coin tossing. The rest only mentioned randomness in words. Two studies were divided into groups based on the sequence of hospital visits and treatment methods, so they were scored as high risk. Four studies reported that patients signed informed consent, and two studies had undergone ethical review. None of the studies reported the study plan, sample size estimation, or concealment of the randomization plan. It was unclear whether there were other biases in all the studies, as seen in Figure 2.

### 3.3 Efficacy assessment

#### 3.3.1 Effective rate

**FIGURE 3:**
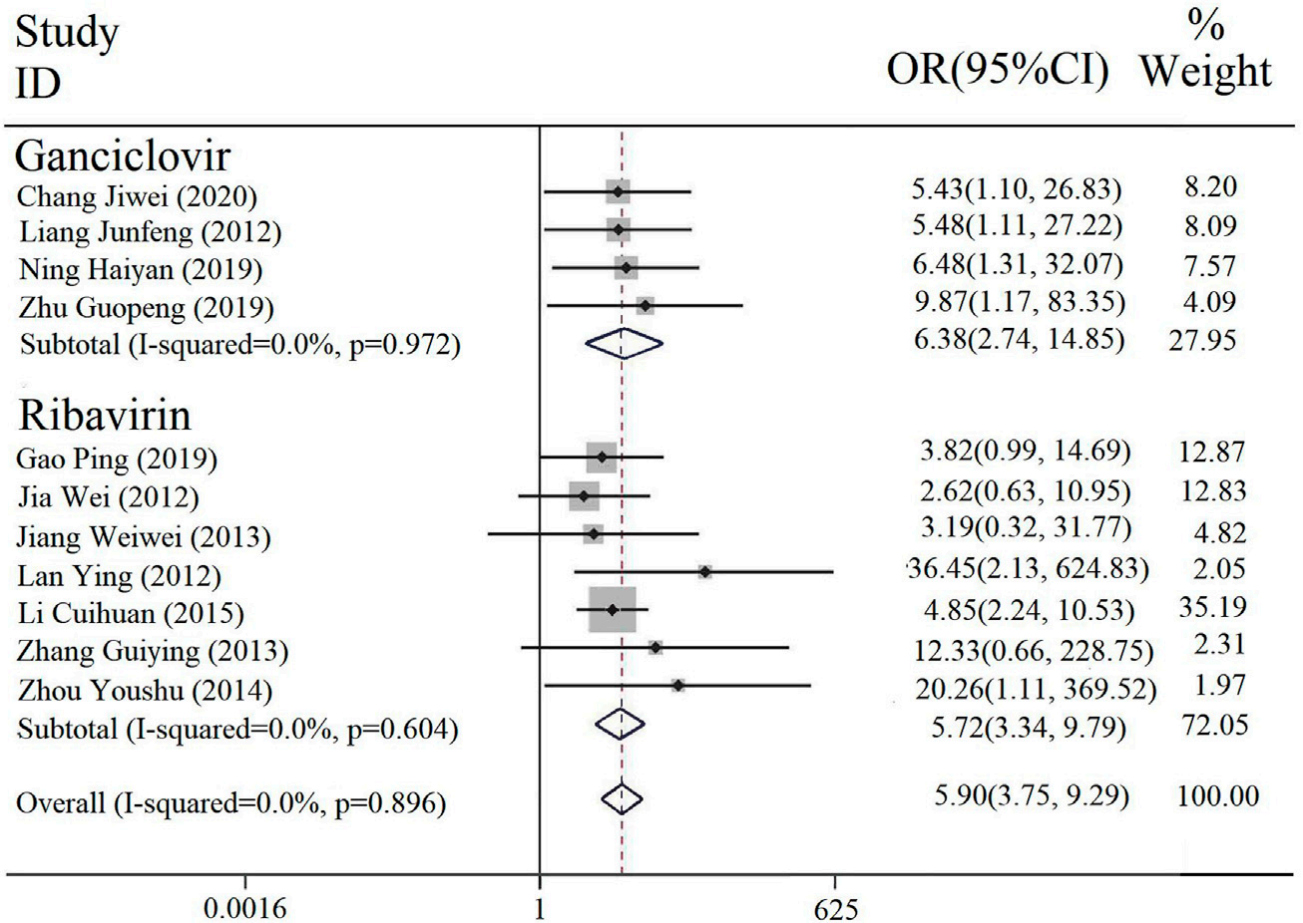
Forest plot of effective rate comparison among groups.

The effective rate was reported in 11 studies, four of which were intervention of PDL combined with ganciclovir (with or without conventional treatment) vs. ganciclovir alone (with or without conventional treatment), and the rest of the seven were intervention of PDL combined with ribavirin (with or without conventional treatment) vs. ribavirin alone (with or without conventional treatment). A subgroup analysis was performed on different interventions, and the heterogeneity results among the subgroups were [*p* = 0.972, I^2^ = 0; *p* = 0.604, I^2^ = 0], and the fixed-effect model was used. The results illustrated that the effective rate of the combined treatment of PDL and ribavirin or ganciclovir was significantly higher than that of ribavirin or ganciclovir [OR = 5.90, 95% CI (3.75, 9.29), P < 0.00001] (Figure 3).

#### 3.3.2 Duration of fever

**FIGURE 4:**
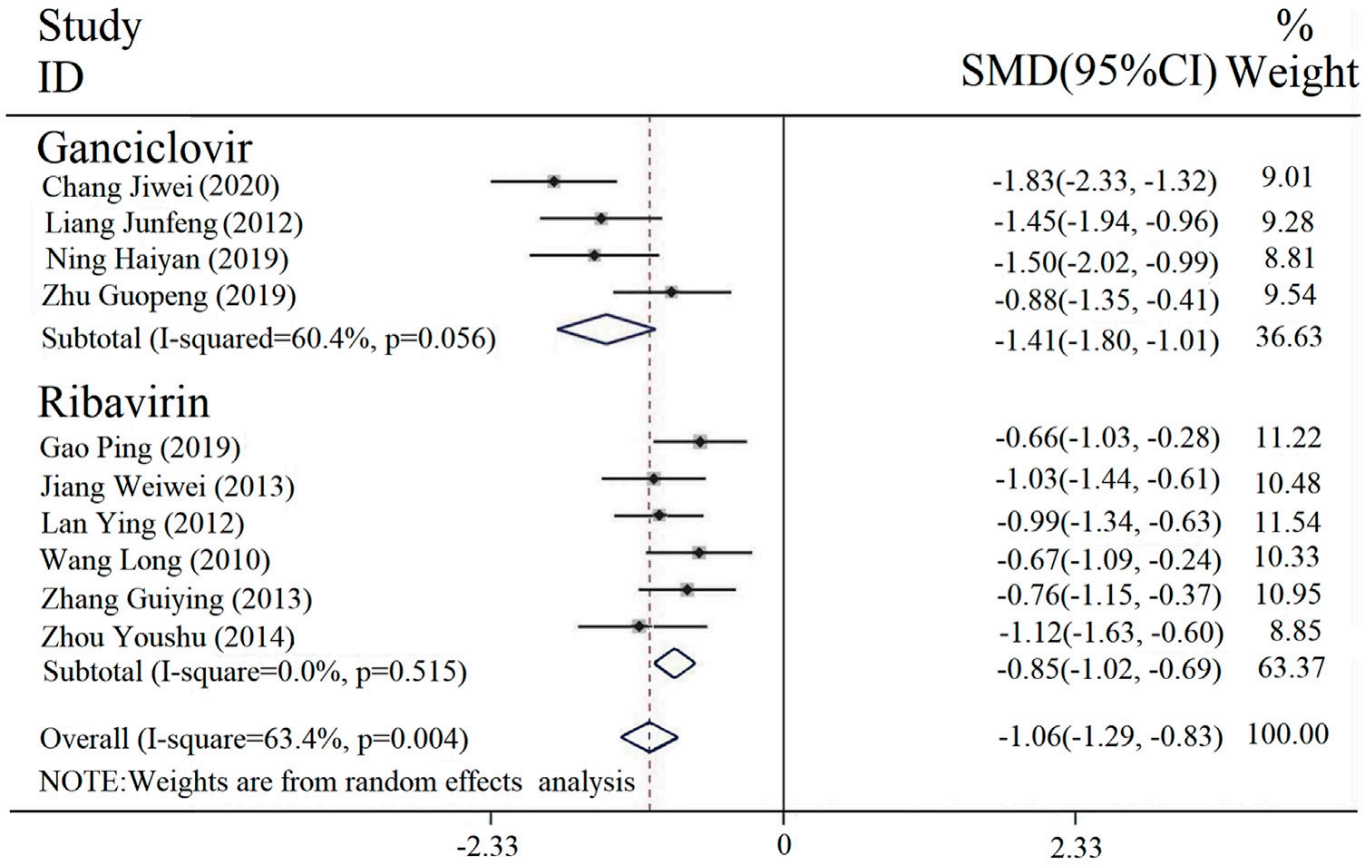
Forest plot of duration of fever comparison among groups.

Duration of fever was reported in 10 studies. The result of the heterogeneity assessment was [Chi^2^ = 23.99, df = 9 (*p* = 0.004), I^2^ = 63.4%], so a random-effect model was incorporated. The results of the meta-analysis (Figure 4) revealed that there was a statistically significant difference in the fever duration between the intervention and control groups [SMD = −1.06, 95% CI (−1.29, −0.83), *p* < 0.00001]. Thus, the combined application of PDL based on antiviral drug treatment (with or without conventional treatment) can relieve fever.

#### 3.3.3 Duration of headache

**FIGURE 5:**
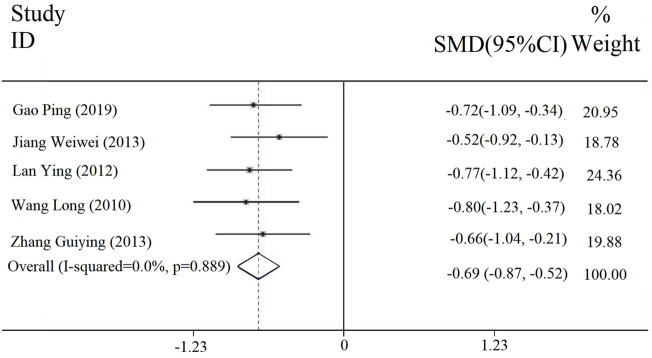
Forest plot of duration of the headache.

Five studies reported the duration of headache, and the heterogeneity results were [Chi^2^ = 1.12, df = 4 (*p* = 0.889); I^2^ = 0], so we involved a fixed-effect model. The meta-analysis (Figure 5) showed that there was a statistically significant difference between the experimental and the control groups in the duration of the headache [SMD = −0.69, 95% CI (−0.87, −0.52), *p* < 0.00001], which indicated that the combined application of PDL with antiviral drug treatment (with or without conventional treatment) could relieve headache.

#### 3.3.4 Duration of parotid gland swelling

**FIGURE 6:**
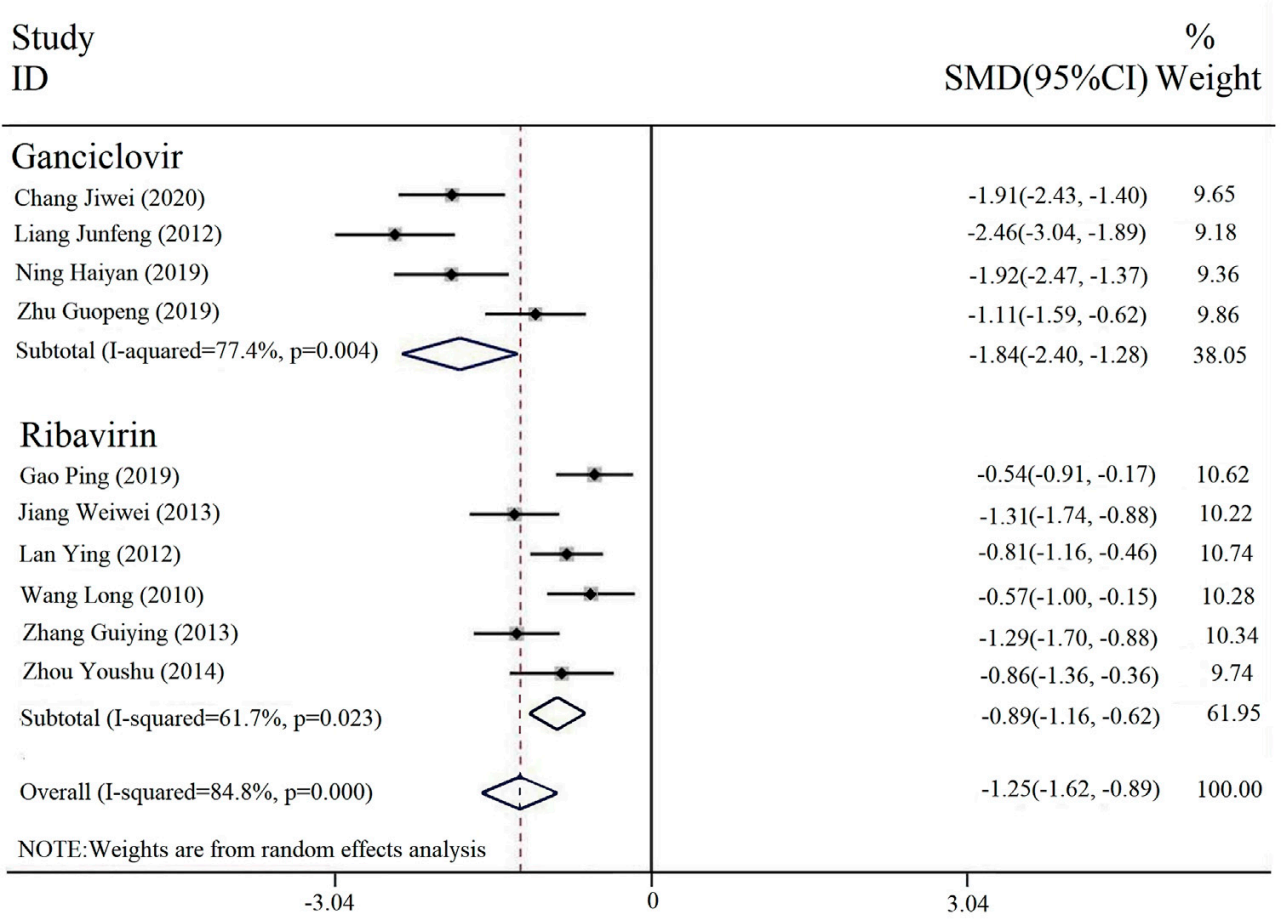
Forest plot of duration of the parotid gland swelling.

Ten of the included studies reported the duration of parotid gland swelling, and four were interventions comparing PDL and ganciclovir (with or without conventional treatment) vs. ganciclovir (with or without conventional treatment). Six of them were interventions combined with PDL and ribavirin (with or without conventional treatment) vs. ribavirin (with or without conventional therapy). The subgroup analysis was conducted on different interventions, and the heterogeneity results among the subgroups were [*p* = 0.004, I^2^ = 77.4%; *p* = 0.023, I^2^ = 61.7%]. A random effect model was selected for analysis. The results indicated that the duration of parotid gland swelling of the combined treatment of PDL and ribavirin or ganciclovir was significantly less than that of ribavirin or ganciclovir alone [SMD = -1.25, 95% CI (-1.62, -0.89), P < 0.00001] (Figure 6).

#### 3.3.5 Duration of parotid gland pain

**FIGURE 7:**
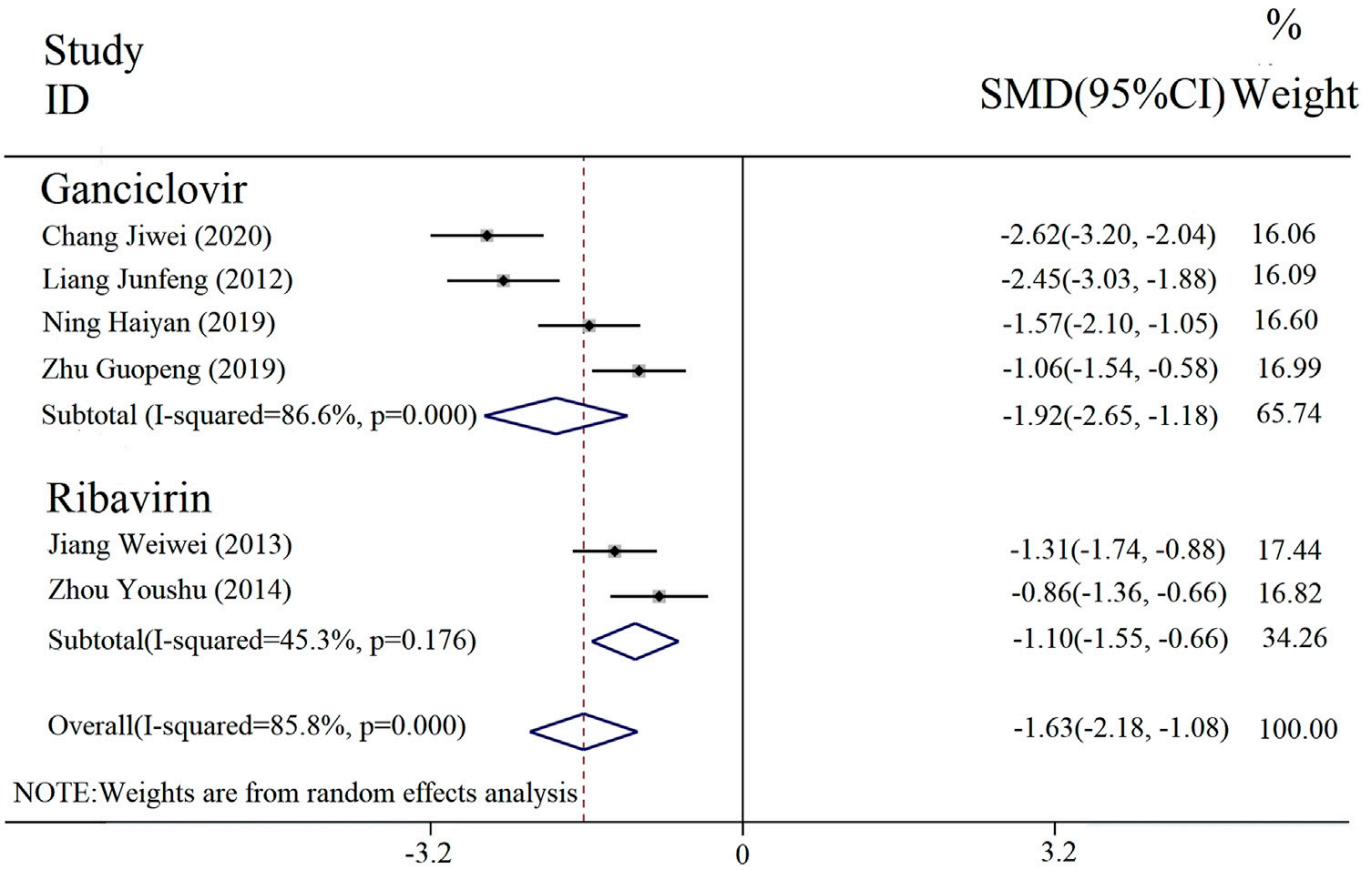
Forest plot of the duration of comparing the parotid gland pain among the groups.

The duration of parotid gland pain was reported in six included studies. The results of the heterogeneity analysis among subgroups divided by different interventions were [*p* = 0.000, I^2^ = 86.6%; *p* = 0.176, I^2^ = 45.3%], and thus a random effect model was incorporated. The results of the meta-analysis (Figure 7) revealed that there was a statistically significant difference in the duration of parotid gland pain between the experimental and control groups [SMD = −1.63, 95% CI (−2.18, −1.08), *p* < 0.000 01]. Thus, the combined applications of PDL based on antiviral drug treatment (with or without conventional treatment) can better relieve parotid gland pain.

#### 3.3.6 Duration of loss of appetite

**FIGURE 8:**
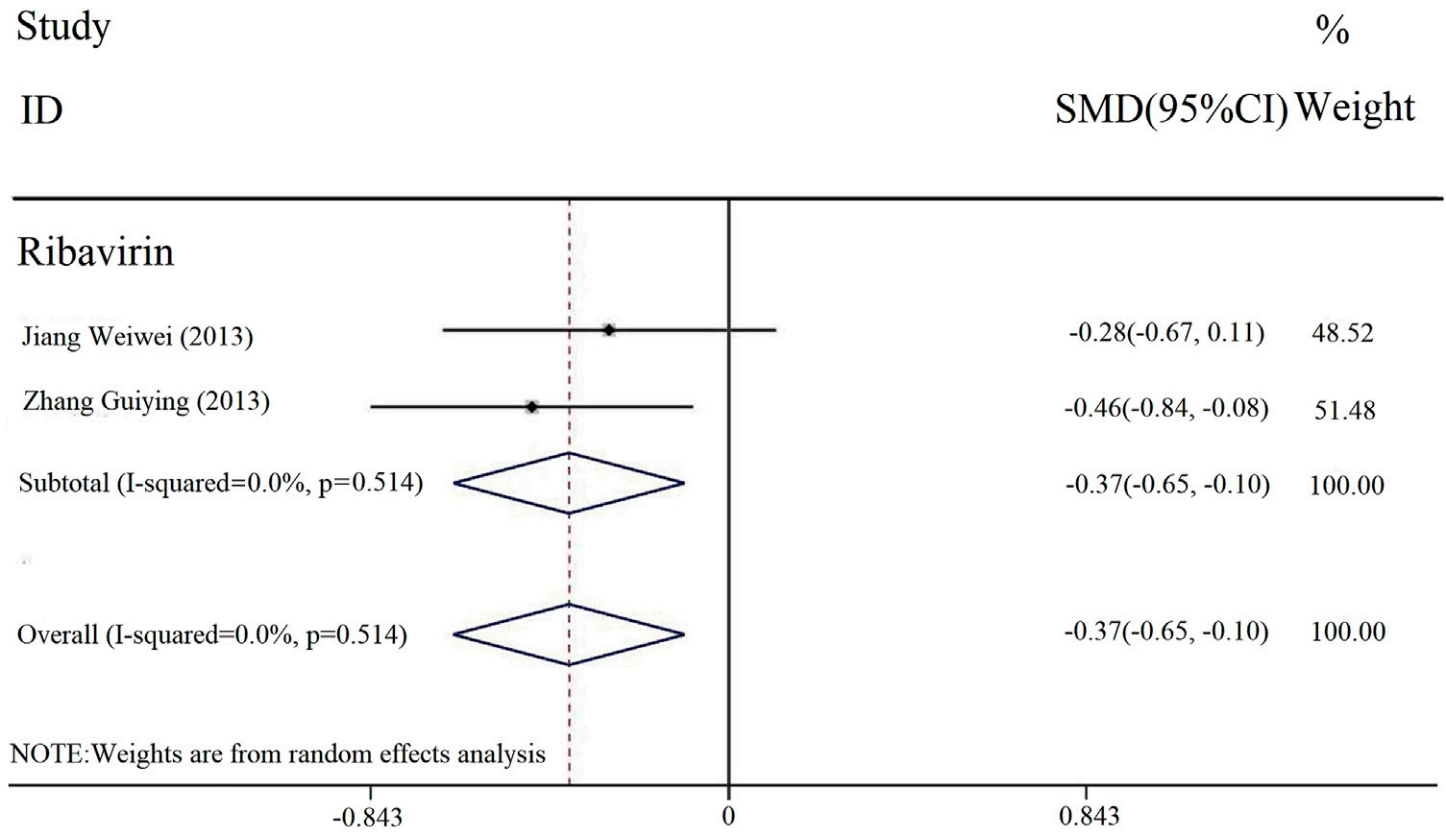
Forest plot of duration of appetite symptom.

The duration of loss of appetite was reported in two studies. The heterogeneity test results were [Chi^2^ = 0.26, df = 1 (*p* = 0.514); I^2^ = 0], and we selected the fixed-effect model. The meta-analysis results (Figure 8) showed that there was a statistically significant difference in the duration of parotid gland pain between the experimental and control groups [SMD = −0.56, 95% CI (−0.96, −0.16), *p* < 0.00001]. It meant that the combined application of PDL based on antiviral drug treatment (with or without conventional treatment) could relieve loss of appetite.

#### 3.3.7 Other outcomes

In addition, three studies compared serum inflammatory factor levels, duration of sore throat, length of hospital stay, and cost of medical care between treatment and control groups, respectively. However, since these outcome indicators were all reported in only one study, we could not conduct a meta-analysis. Nevertheless, it could still be seen from the results that PDL combined with antiviral drugs reduced inflammation, sore throat, length of hospital stay, and cost of medical care in children more than using one antiviral drug alone for mumps (Table 2).

**TABLE 2:** Other outcomes.

Study	Year	N	Outcome indicator	T		C		P	
		T/C		Before	After	Before	After	P1	P2
Lan Ying	2012	68/68	Duration of sore throat	5.0 ± 2.6		7.0 ± 3.0		<0.01	
Ning Haiyan	2019	37/37	CRP (mg/L)	15.54 ± 3.21	6.39 ± 1.91	15.31 ± 3.30	7.88 ± 2.46	0.76	0.00
			IL6 (mg/L)	118.97 ± 16.27	82.87 ± 9.49	121.52 ± 15.19	97.23 ± 13.46	0.49	0.00
			TNF-α (pg/ml)	892.17 ± 60.20	463.34 ± 37.49	897.30 ± 62.31	621.85 ± 50.38	0.72	0.00
Zhu Guopeng	2019	38/38	Length of hospital stay	6.75 ± 2.26		9.75 ± 2.19		0.002	
			Cost of medical care	3.90 ± 1.48		5.18 ± 1.37		0.031	

Note: T: test group; C: control group; P1: *p*-value of the comparison between the control and the treatment groups before intervention; P2: *p*-value of the comparison between the control and the treatment groups after intervention.

### 3.4 Adverse reactions

Four studies (Jia, 2012; Li, 2015; Zhu, 2019; Chang, 2020) reported adverse reactions, including diarrhea, abdominal pain, oral ulcers, rash, neutropenia, anemia, and constipation. All the clinical symptoms and signs were transient without specific treatment and did not cause adverse consequences. As for the remaining eight studies, no adverse reactions or events were reported.

### 3.5 Publication bias

**FIGURE 9:**
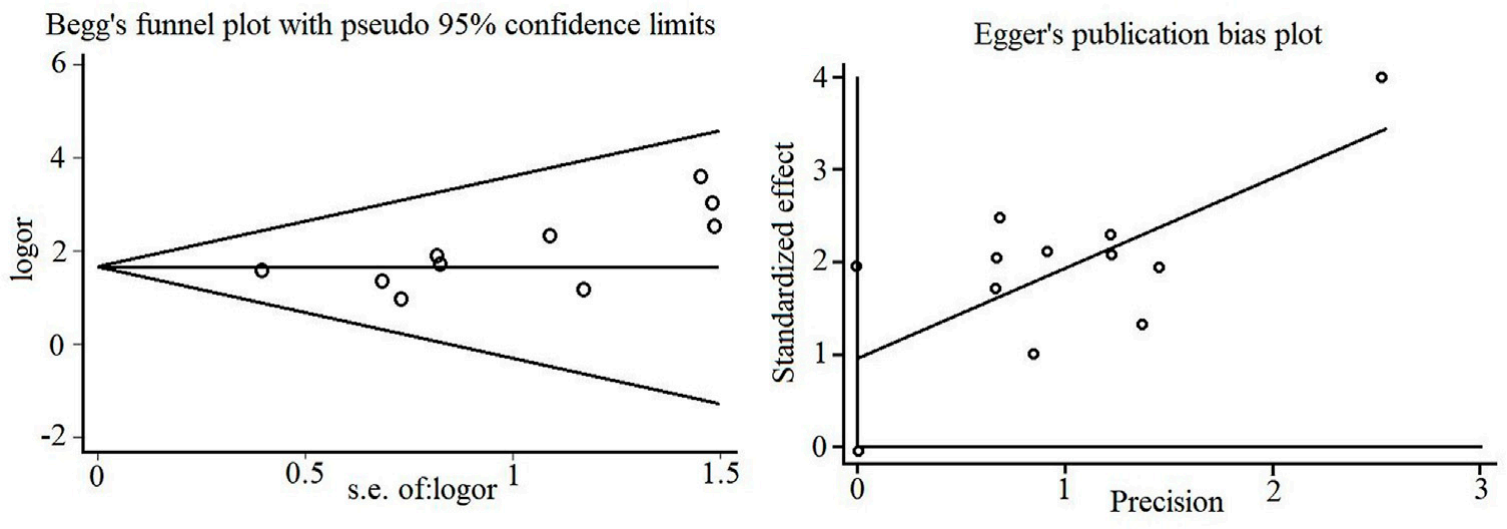
Begg’s and Egger’s funnel plots for publication bias.

The effective rate of the included studies was utilized as the data to detect publication bias and generate Begg’s and Egger’s funnel plots, as shown in Figure 9. Egger’s test revealed a certain degree of publication bias (*p* = 0.045).

### 3.6 Sensitivity analysis

**FIGURE 10:**
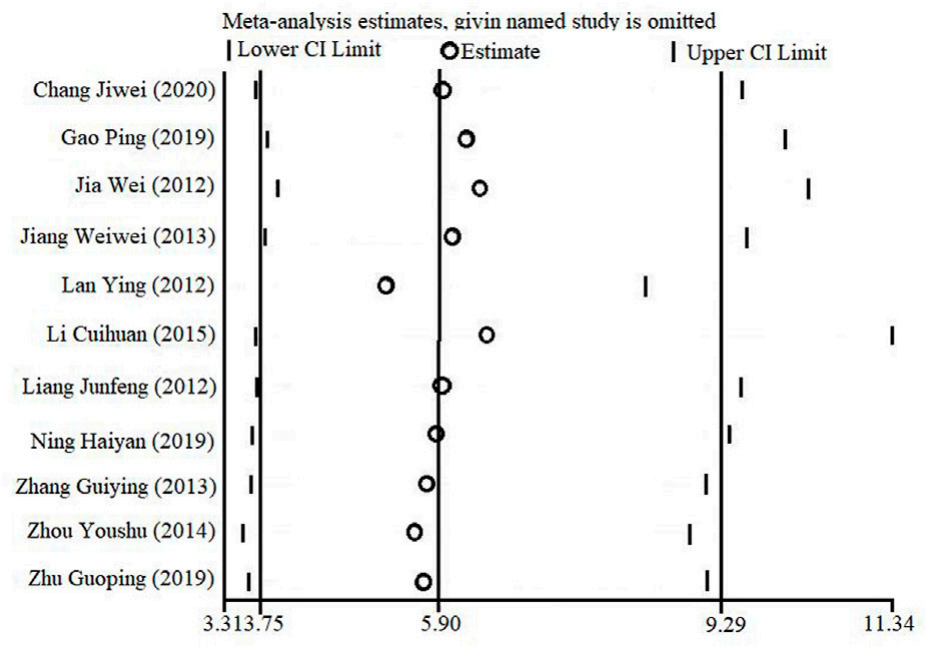
Results of sensitivity analysis.

With the one-by-one exclusion method, a sensitivity analysis based on STATA software was performed to evaluate the overall combined effect quantity of the effective clinical rate outcome index. That is, one independent study was excluded each time, and the remaining studies were re-analyzed to determine the stability of the effect size. As shown in Figure 10, the combined effect quantity did not change qualitatively, suggesting that the results of this study were stable.

### 3.7 Collection of components and targets in PDL

We searched the TCMSP database and CNKI reviews (Nie et al., 2020; Wu et al., 2020), combined with SwissADME and SwissTargetPrediction, to collect the active components and the related targets of PDL. After deduplication and exclusion, a total of 119 active components, five repeating components, and 480 associated targets were acquired. The specific component information is represented in Supplementary Table S2.

### 3.8 Collection of targets of mumps

**FIGURE 11:**
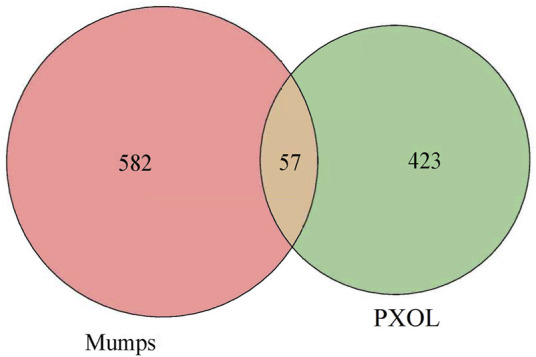
Mumps–PDL intersection targets.

The mumps-related targets were not obtained by retrieving the databases of GEO and TTD, while 694 and 79 targets were obtained from Genecards and OMIM, respectively. After processing the abovementioned targets, 639 mumps-related targets were obtained. Subsequently, Excel was used to intersect the drug and disease targets and received 57 intersecting targets directly related to drugs and diseases (Figure 11).

### 3.9 Analysis of “drug-component-target” network

**FIGURE 12:**
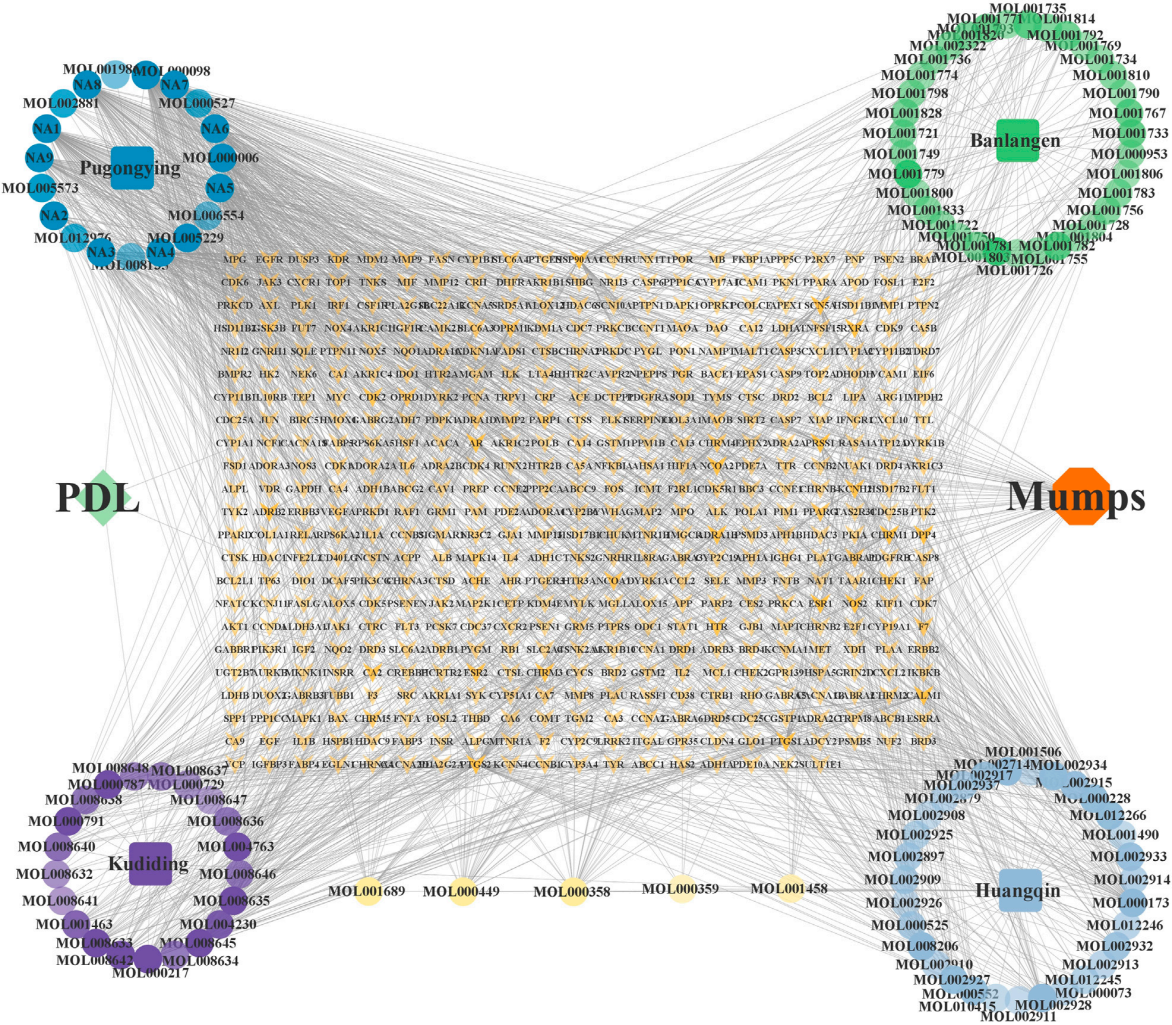
Drug-component-target network.

The “drug-component-target” network, having 597 nodes and 1,914 edges, was constructed after importing the correlation data within the Cytoscape software, as shown in Figure 12. From the network, 11 core active components having node degree ≥40 were screened out, including quercetin, isoetin, taraxacin, ethyl p-hydroxyphenylacetate, 11β, 13-dihydrotaraxinic acid, chlorantholide C, artecalin, luteoli, methyl p-hydroxyphenylacetate, wogonin, and arsanin.

### 3.10 Analysis of PPI network

**FIGURE 13:**
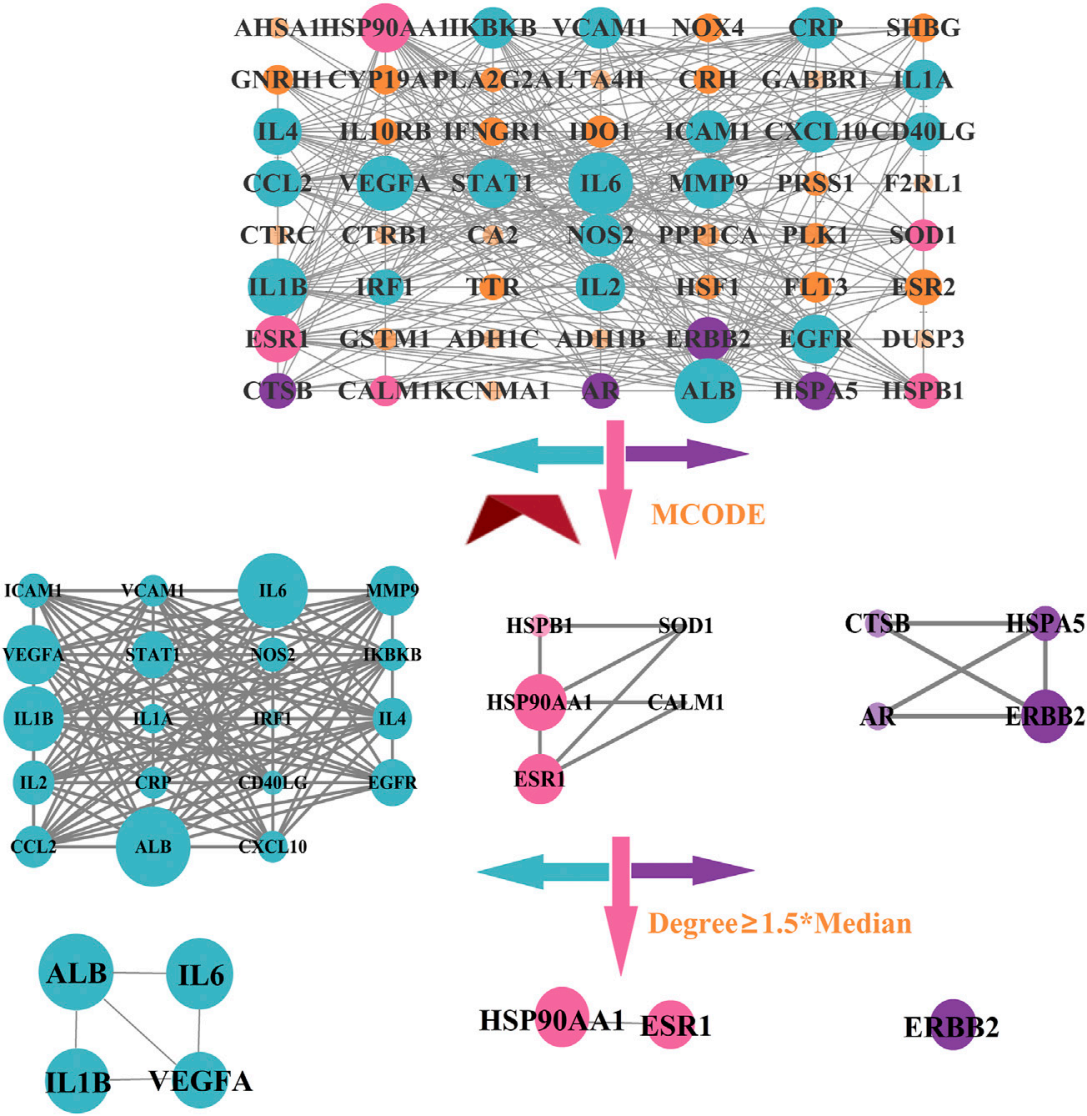
PPI network of intersection targets.

A PPI network in which the number of nodes was 57, the number of edges was 391, the average node degree was 13.7, and the *p*-value for PPI clustering was less than 1.0e-16 was constructed after importing the intersection targets into STRING and Cytoscape. Then, the MCODE plugin was used to cluster the PPI network, and three clustering networks were obtained, as shown in Figure 13. Finally, we selected the targets having node degree ≥1.5*median in each clustering network as the core targets, such as albumin (ALB), interleukin-6 (IL6), interleukin-1 beta (IL1B), vascular endothelial growth factor A (VEGFA), heat shock protein HSP 90-alpha (HSP90AA1), estrogen receptor (ESR1), and the receptor tyrosine-protein kinase erbB-2 (ERBB2).

#### 3.11 Enrichment analysis of core intersection targets

**FIGURE 14:**
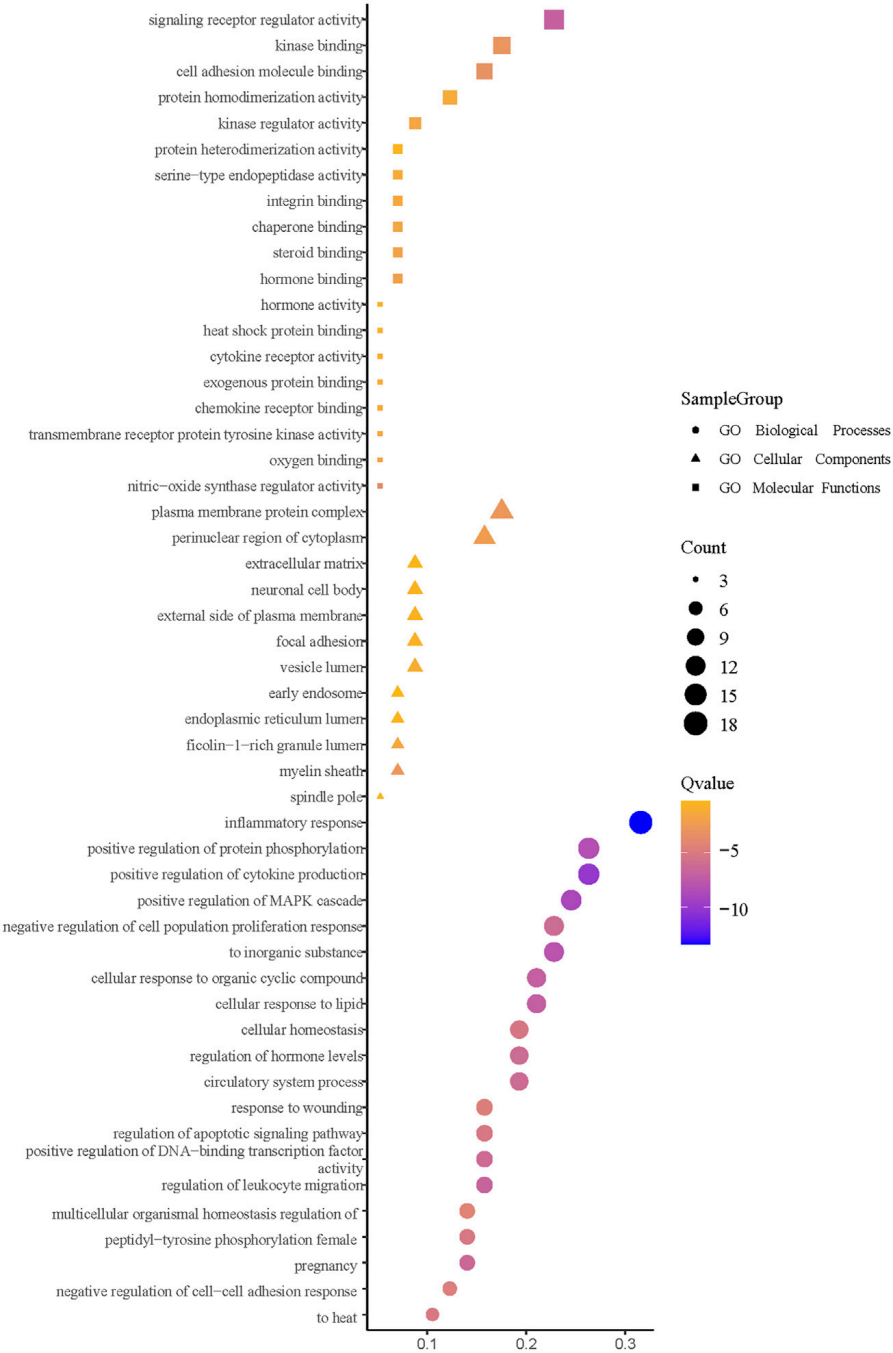
GO enrichment analysis.

**FIGURE 15:**
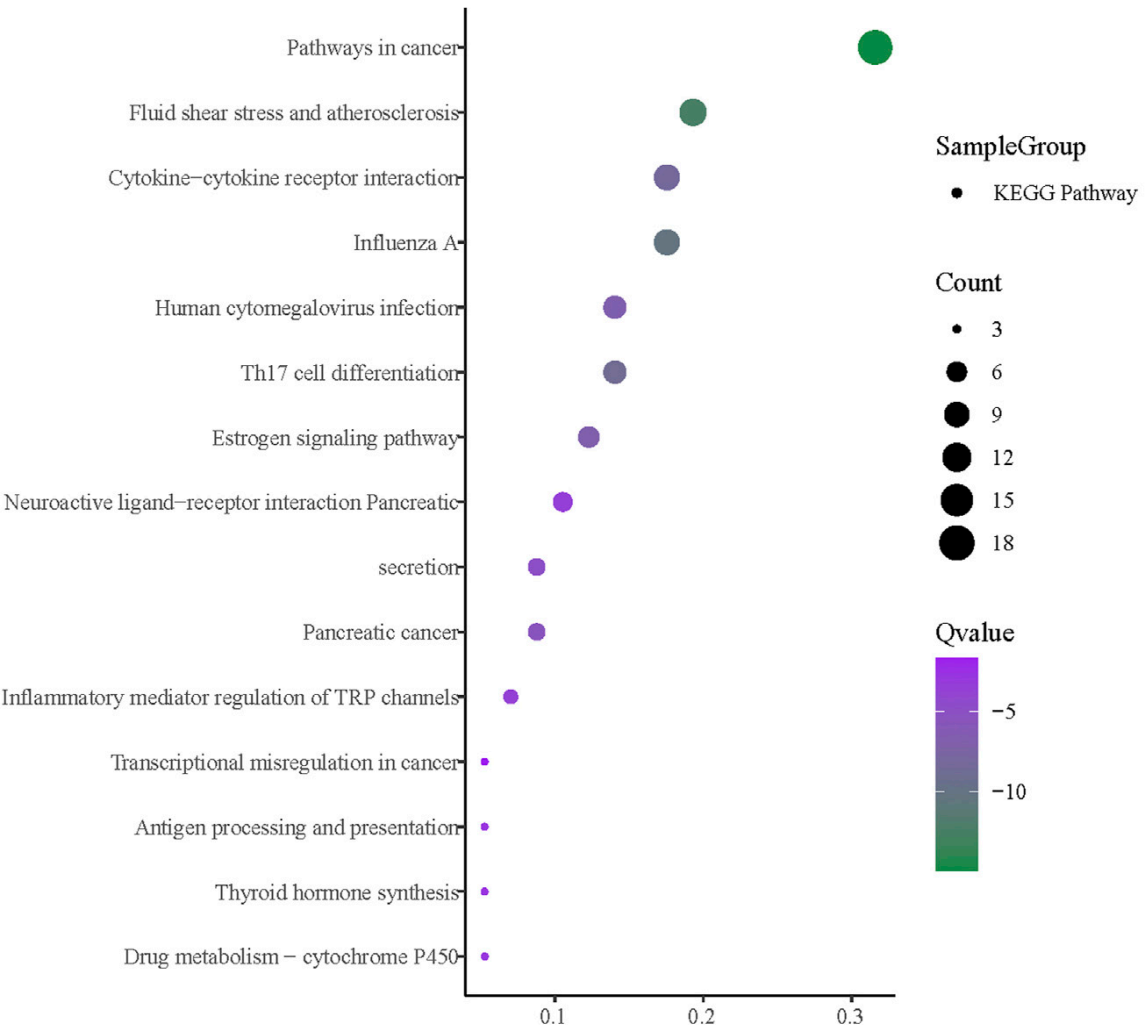
KEGG enrichment analysis.

The enrichment analysis was facilitated by importing the intersection targets into Metascape (Figure 14). The available GO biological processes included inflammatory response, positive regulation of cytokine production, positive regulation of mitogen-activated protein kinase (MAPK) cascade, positive regulation of protein phosphorylation, and response to an inorganic substance. The GO molecular functions included signaling receptor regulator activity, nitric-oxide synthase regulator activity, and cell molecule binding. Moreover, the GO cell components had a plasma membrane protein complex, myelin sheath, and perinuclear region of the cytoplasm. KEGG pathway enrichment analysis is depicted in Figure 15. It can be seen that the primary ones are pathways in cancer, fluid shear stress, atherosclerosis, influenza A, Th17 cell differentiation, and cytokine–cytokine receptor interaction.

### 3.12 Molecular docking analysis

**FIGURE 16:**
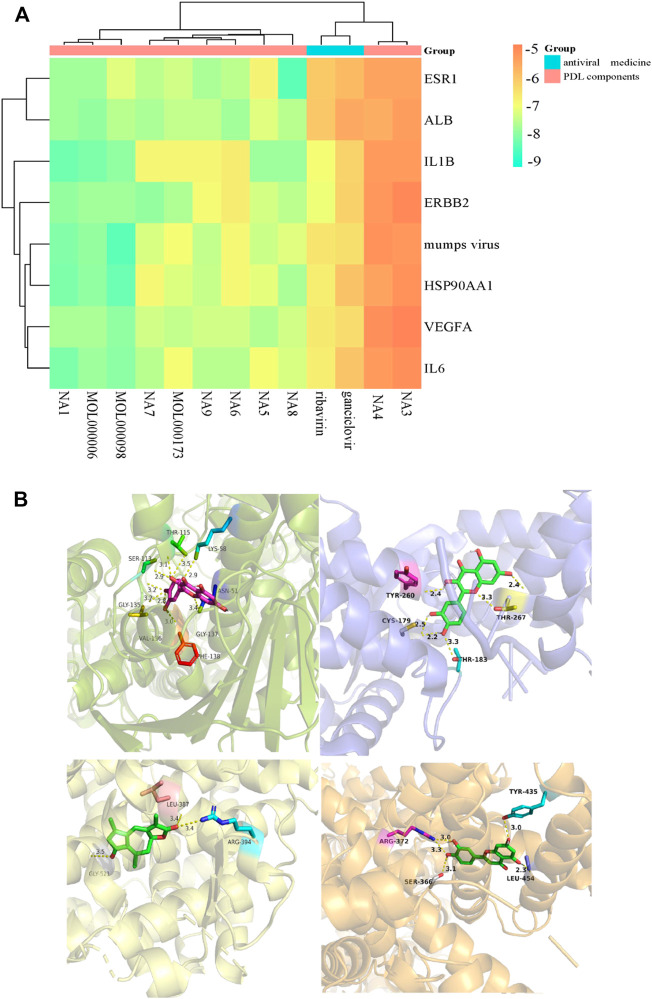
**(A)** Heat map of the molecular docking results between core components and targets; **(B)** docking models of the best combinations (from top left to bottom right: quercetin and HSP90AA1, taraxacin and ESR1, quercetin and MuV, and luteolin and ALB).

We obtained that most docking combinations had binding energy lower than -5.0 kcal/mol by performing molecular docking on the core targets and components, setting the most common control drugs (ribavirin and ganciclovir) and the MuV as controls. It suggested that most of the core components and the core targets could form stable structures. In contrast, the docking results of ribavirin and ganciclovir indicated that they were only better than methyl p-hydroxyphenylacetate and ethyl p-hydroxyphenylacetate. In addition, the docking results of MuV revealed that all core components could form stable binding with it. See Figure 16A, Supplementary Table S3, and Supplementary Table S4 for details such as binding energy, and the best docking combinations are represented in Figure 16B. It could be seen that quercetin was strongly targeted with THR-115, LYS-58, ASN-51, GLY-137, PHE-138, VAL-136, GLY-135, and SER-113 residues of HSP99AA1 by hydrogen bonding with the docking energy = -9.9 kcal/mol. ESR1 interacted with taraxacin through hydrogen bonding, associating taraxacin and TYR-260, THR-267, THR-183, and CYS-179 with a docking energy of -9.2 kcal/mol. Moreover, quercetin was targeted with residues including LEU-387, ARG-394, and GLY-521 of MuV by hydrogen bonding in which the docking energy is -8.6 kcal/mol. ALB could also bind strongly with the residues, including TYR-435, LEU-454, ARG-372, and SER-366 of luteolin by hydrogen bonding with the binding energy = -8.4 kcal/mol.

## 4 Discussion

Mumps is from the category of “Zhasai” in TCM. From the perspective of TCM, this disease is characterized by the invasion of the human body from the mouth and nose through wind, warm, and toxic pathogens. Moreover, heat accumulation in the liver and gallbladder inside the body blocks the Shaoyang meridians, which leads to the circulation of qi and blood. Qi and blood are stagnated inside the parotid and cheek, causing non-suppurative swelling and pain centered on the earlobe (Zhou, 1987). Therefore, TCM is focused on the perspective of clearing away heat and toxic materials for its treatment. The four herbs in PDL exert these effects.

The results of our study demonstrated that the combinations of PDL and antiviral drugs in the treatment of mumps could improve the efficacy and relieve related symptoms such as fever, headache, parotid gland swelling, parotid pain, and loss of appetite more rapidly than using antiviral drugs such as ganciclovir and ribavirin alone (with or without conventional treatment). The use of PDL can benefit children with mumps, reduce treatment time and costs, and alleviate discomfort, which has more significant implications for pediatric patients and society.

Of the 12 included studies, only four reported adverse reactions. Although the adverse reactions did not affect the treatment, were tolerated by the patients, or disappeared after drug withdrawal, the side effects of PDL are not evident in its instructions. Therefore, researchers need to record the occurrences of adverse reactions/events. The efficacy of traditional Chinese medicine should be guided by reasonable syndrome differentiation. However, PDL is widely used in clinical practice in traditional Chinese medicine hospitals and in many western medicine hospitals and community hospitals to treat mumps. Moreover, patients can purchase it alone, which could cause inaccurate syndrome differentiation. Therefore, when using PDL, doctors should actively observe the patient’s response to the drug under the premise of correct syndrome differentiation.

Only three included studies reported specific randomization methods (coin toss or random number table), and the rest only mentioned randomization in words. Two studies with high risk are grouped by a different order of visits or medication methods. It was uncertain whether randomization, blinding, and allocation concealment were performed, so there could be implementation and measurement bias. None of the included RCTs mentioned using double-blinding. However, because the blinding method can effectively evaluate the treatment effect and the quality of blinding will directly affect the accuracy of the research, it is suggested that relevant future studies should pay attention to the implementation of randomization schemes, concealment schemes, and blinding methods during the design and specific operation of trials.

Moreover, the efficacy evaluation indicators are not comprehensive, such as virus infection, inflammatory, long-term efficacy, and long-term outcomes, including recurrence and complication rates, were not reported. Although the adverse side effects described are few, and most of them are not directly associated with the use of PDL, the safety of PDL in treating mumps still needs further discussion combined with long-term indicators. The sample size of each included study was small. Only one study had a sample size of more than 100 cases, and the rest had a sample size of less than 100. The efficacy indicators of these studies with small sample sizes could not be stable. The results of Begg’s and Egger’s tests indicated the existence of publication bias, and the subjective bias of the researchers in publishing the results could exaggerate the effect of the experimental group in improving the symptoms of mumps.

Based on the meta-analysis, PDL associated with conventional treatment in treating children’s mumps could better relieve symptoms and physical signs with fewer adverse reactions. It has been suggested that PDL can be added based on conventional therapy and antiviral drugs in the clinical treatment of mumps. In addition, this study conducted a subgroup analysis of the antiviral drugs commonly used in the treatment of mumps, which made it clear and pertinent. Some of the studies were published earlier, and some were of poor methodological quality, which would have had a significant impact on the reliability of the conclusions. Therefore, we suggest that clinicians refer to the results of this study for the clinical application of PDL on the premise of combining their clinical diagnosis, treatment experience, and clinical conditions.

Among the various core components derived from network pharmacology: quercetin has been reported to inhibit different viruses (including influenza A virus, SARS-CoV-2, and pseudorabies virus) and exhibits antiviral and anti-inflammatory effects (Li et al., 2016; Sun Y. et al., 2021; Saeedi-Boroujeni and Mahmoudiani, 2021); luteolin has been found to inhibit certain viruses including influenza A virus, respiratory syncytial virus, and SARS-CoV-2 by interfering with viral replication and virus-related protein expression. Moreover, luteolin and some of its derivatives could also regulate immunity and inflammation by regulating the transcription factors such as signal transducer and activator of transcription 3 (STAT3), nuclear factor kappa-B (NF-κB), and activator protein 1 (AP-1) (Aziz et al., 2018; Yan et al., 2019; Wang et al., 2020); wogonin is an active component obtained from the Chinese herbal medicine baical skullcap root, confirmed to have antiviral (herpes simplex virus, hepatitis B virus, and coronavirus), antibacterial, anti-inflammatory, and immune regulatory effects (Guo et al., 2007; Chu et al., 2020; Huang et al., 2020; Hyunh et al., 2020). Although the abovementioned components have not been directly analyzed to reveal their therapeutic effects on mumps or their inhibitory effects against the mumps virus, it can be seen from previous studies that they possess broad-spectrum antiviral effects and can relieve the associated symptoms by regulating immunity and inflammation. Therefore, they promote antiviral effects, relieve symptoms, and may have the same effects on mumps. For the other core components, no study has reported their specific pharmacological effects. Thus, our study can provide their research directions to a certain extent.

In the clusters of targets, cluster 1 mainly targets immunity and inflammation. Among them, ALB is a multifunctional protein within the human serum. Its functions involve binding activity with toxic substances, negative regulation of apoptosis, and other immune-related roles (National Center for Biotechnology Information, 2022). IL6 and IL1B, as immune-related cytokines, could mediate the immune response of mumps and have regulatory effects on mumps complications such as meningitis and orchitis (Asano et al., 2011; Wu et al., 2016; Riggenbach et al., 2022). VEGFA encodes an angiogenesis-related binding protein. Infection with some viruses (e.g., SARS-CoV-2 and dobrava/belgrade virus) increases its expression and promotes inflammation (Tsergouli and Papa, 2013; National Center for Biotechnology Information, 2022). Among the targets in cluster 2, HSP90AA1 mainly acts as a molecular chaperone, promoting maturation, structural maintenance, and proper regulation of specific target proteins. Moreover, it plays a role in the transcriptional machinery to enhance host antiviral responses (National Center for Biotechnology Information, 2022). ESR1 encodes the type 1 estrogen receptor, and several reports show that the incidence of mumps in male children is significantly higher than in females (Hassan et al., 2012). However, the mechanism is related to ESR1 is still unknown, and thus further research is needed. Cluster 3 has one core target of ERBB2, which mainly has receptor binding and enzyme binding activity. It participates in the positive regulation of macromolecular biosynthesis, protein phosphorylation, and MAPK cascade regulation. It can also induce inflammation in cancer (Zeng et al., 2012; National Center for Biotechnology Information, 2022), but its role in viral infection remains unknown. Therefore, we can understand from the core targets that the therapeutic effects of PDL on mumps primarily relieve inflammation and symptoms through immune effects.

The GO enrichment analysis shows that the main results are also related to immunity and inflammation. For KEGG enrichment analysis, the pathways in cancer could suggest a relationship between cancer and mumps. For instance, a study by Cramer et al. (2010) showed that mumps might create effective immune surveillance of ovarian cancer cells, partly explaining the preventive effect of children’s mumps on ovarian cancer. In addition, a study by Ammayappan et al. (2016) revealed that the mumps virus has therapeutic potential for various cancers and showed therapeutic efficacy in animal tumor models. The result of fluid shear stress and atherosclerosis indicates that there could be a relationship between cardiovascular and mumps infection. Thus, a questionnaire study by Kubota et al. (2015) revealed that mumps infection could reduce atherosclerosis mortality. Additionally, immune and inflammation-related pathways, including influenza A, Th17 cell differentiation, cytokine–cytokine receptor interaction, and estrogen signaling pathways, have been discussed previously.

Finally, on the one hand, the molecular docking results revealed that most of the combinations of the core components and core targets could form stable structures. Cell experiments have confirmed that quercetin could reduce IL6 and IL1β, exhibiting anti-inflammatory and antioxidative stress effects (Li et al., 2021). In contrast, low concentrations of quercetin could downregulate the expression of VEGFA and matrix metalloproteinase-9 (MMP9). Matrix metalloproteinase-2 (MMP2) mediates migration and angiogenesis inhibition (Liu et al., 2017). Since elevated VEGFA can increase inflammation, quercetin can reduce inflammation by decreasing VEGFA. Luteolin can inhibit the NF-κB cellular pathway to stimulate gene expression, encoding inducible pro-inflammatory enzymes and cytokines, including IL-1β, IL6, and TNF-α. Thus, it exhibits strong anti-inflammatory effects (Mahdiani et al., 2022). A study (Pratheeshkumar et al., 2012) used *in vitro*, *ex vivo*, and *in vivo* models to explore the antiangiogenic activity of luteolin, and the results revealed that luteolin could cause a dose-dependent and statistically significant reduction in VEGF secretion. Moreover, wogonin has been observed to inhibit the production of nitric oxide (NO) and the expression of IL-1β, IL-6, TNF-α, and VEGF in macrophages activated through virus-mimicking double-stranded RNA, thereby exhibiting immunosuppressive and anti-inflammatory effects (Liao et al., 2021). As for other combinations, no suitable studies could be found, so more research is needed. On the other hand, compared to the two antiviral drugs used as the control group and combined with the meta-analysis, namely, ribavirin and ganciclovir, most of the core components have lower binding energy, forming more stable structures. In addition, all core components include stable structures with MuV. This can explain the material basis of the effective components of PDL to enhance the efficacy of mumps treatment.

This is the first article that integrated meta-analysis and network pharmacology to evaluate the efficacy and possible mechanisms of TCM patent medicine among children with mumps. To an extent, this article made some progress, filled some gaps in this field, and guided our ongoing research. However, this study is mainly based on literature research and databases, so experiments or clinical trials still need to verify the specific conclusions.

## 5 Conclusion

Therefore, the data of included studies and methods of systematic review proved that PDL combined with antiviral drug therapy could improve the effective rate of mumps treatment and better relieve symptoms such as fever, headache, parotid swelling, parotid pain, and loss of appetite in mumps among children with no severe adverse reactions. However, given the quality of the included studies, more high-quality RCTs with large sample sizes, scientific design, multi-center, and strict implementation are needed to discuss the efficacy and safety of PDL in treating mumps to improve the scientific credibility of the literature. Moreover, it could provide a basis for applying PDL in treating mumps and facilitate high-quality evidence support for clinical practice. The network pharmacology study predicted that the therapeutic effects of PDL on mumps are *via* the immunoregulatory effects of quercetin, luteolin, wogonin, and other components on ALB, IL6, IL1A, HSP90AA1, ERBB2, and other targets. It can facilitate further research.

## Data Availability

The original contributions presented in the study are included in the article/Supplementary Material; further inquiries can be directed to the corresponding author.

## Abbreviations

ALB, albumin; AP-1, activator protein 1; CI, confidence interval; CNKI, China National Knowledge Infrastructure; Covid-19, corona virus disease 2019; CRP, C-reactive protein; DL, drug-likeness; ERBB2, receptor tyrosine-protein kinase erbB-2; ESR1, estrogen receptor; GEO, Gene Expression Omnibus; GO, Gene Ontology; HSP90AA1, heat shock protein HSP 90-alpha; IL1B, interleukin-1 beta; IL6, interleukin 6; KEGG, Kyoto Encyclopedia of Genes and Genomes; MAPK, mitogen-activated protein kinase; MMP2, matrix metalloproteinase-2; MMP9, matrix metalloproteinase-9; MuV, mumps virus; NF-κB, nuclear factor kappa-B; NO, nitric oxide OB, oral bioavailability; OMIM, Online Mendelian Inheritance in Man; OR, odds ratio; PPI, protein–protein interaction; PRISMA, Preferred Reporting Items for Systematic Reviews and Meta-Analyses; PROSPERO, International Prospective Register of Preferred Reporting Items; PDL, Pudilan Xiaoyan oral liquid; RCT, randomized controlled trials; SARS-CoV-2, severe acute respiratory syndrome coronavirus 2; SMD, standardized mean difference; STAT3, signal transducer and activator of transcription 3; TCM, traditional Chinese medicine; TCMSP, traditional Chinese medicine system pharmacology technology platform; TNF-α, tumor necrosis factor alpha; TTD, Therapeutic Target Database; VEGFA, vascular endothelial growth factor A.
