# Supplementary figures and images for: Clinical efficacy evaluation and potential mechanism prediction on Pudilan Xiaoyan oral liquid in treatment of mumps in children based on meta-analysis, network pharmacology, and molecular docking
Source: Front Pharmacol. 2022 Sep 23;13:956219. doi: 10.3389/fphar.2022.956219 (PMC9537475; doi:10.3389/fphar.2022.956219)

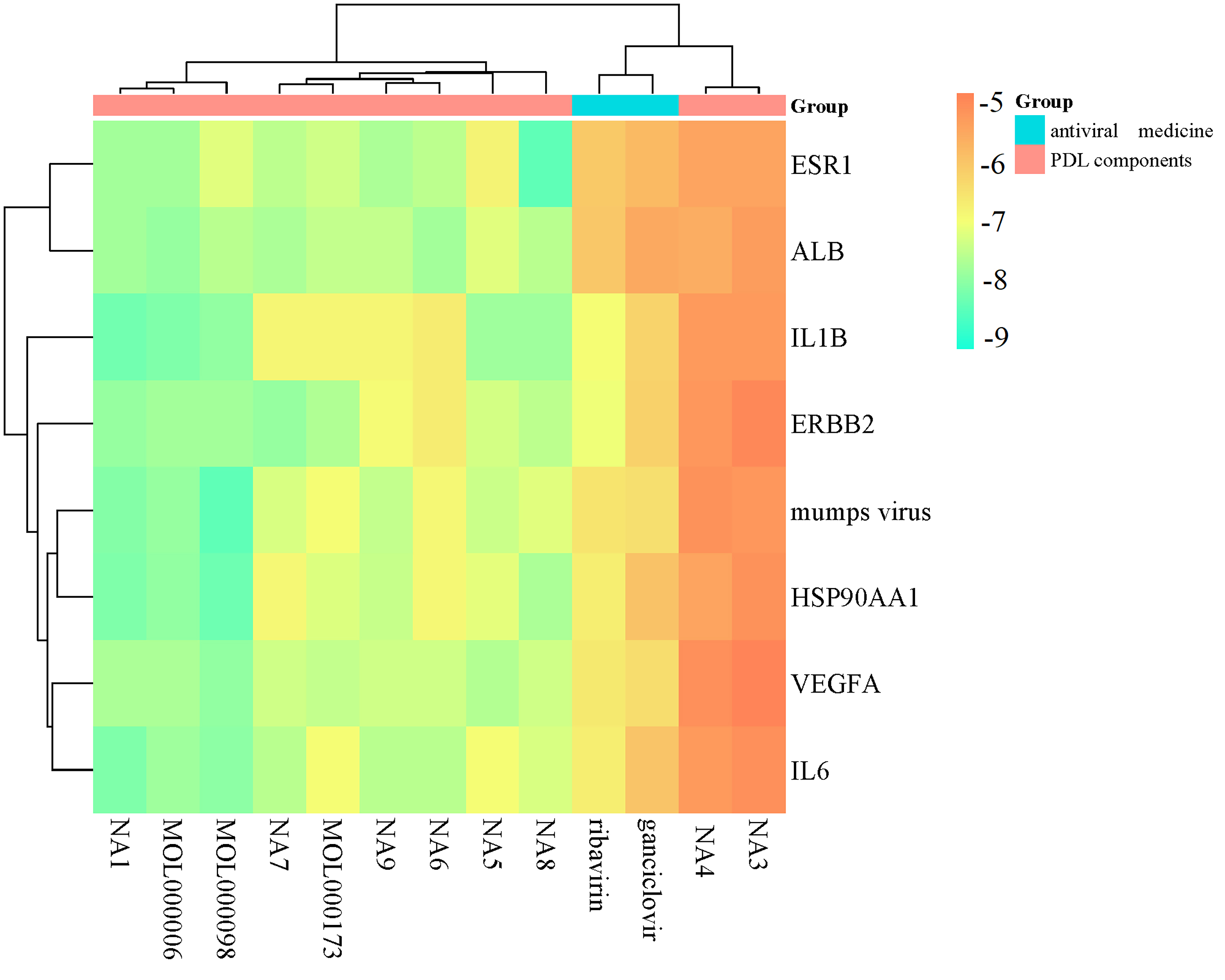

Supplement: Supplementary file 1 [file Image1.JPEG]

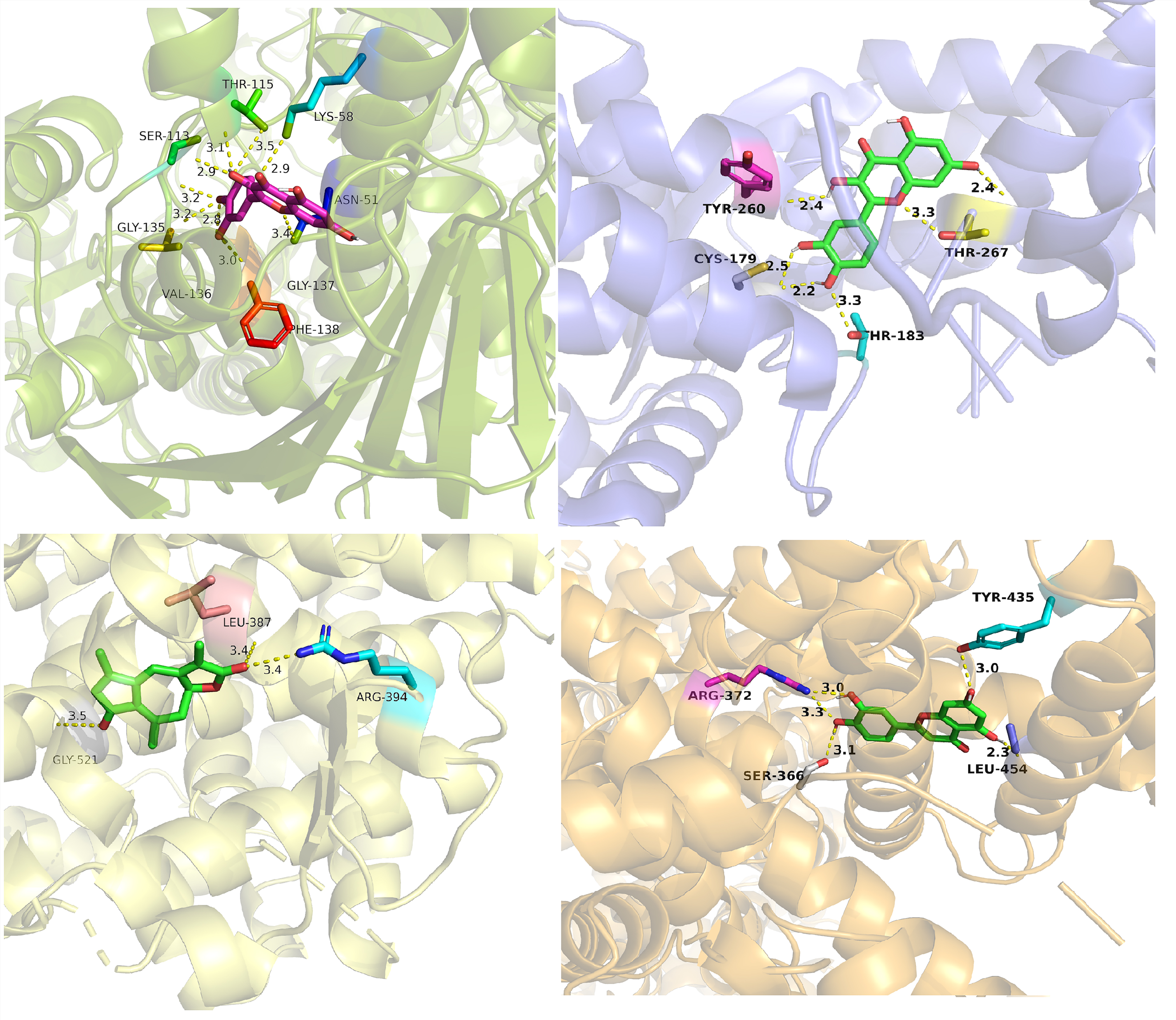

Supplement: Supplementary file 2 [file Image2.JPEG]
