# Supplementary material for: Clinical efficacy evaluation and potential mechanism prediction on Pudilan Xiaoyan oral liquid in treatment of mumps in children based on meta-analysis, network pharmacology, and molecular docking
Source: Front Pharmacol. 2022 Sep 23;13:956219. doi: 10.3389/fphar.2022.956219 (PMC9537475; doi:10.3389/fphar.2022.956219)
Supplement: Supplementary file 3 [file DataSheet1.PDF]

Supplementary Material

Table S1 Search strategies for every databases

Database	Query	#ID	Records
CNKI	(SU=流行性腮腺炎 or SU=痄腮) and (SU=小儿 or SU=儿童 or SU=未成年) and SU=蒲地蓝		13
WanFang	主题("流行性腮腺炎" or "痄腮") and ("小儿" or "儿童" or "未成年") and ("蒲地蓝")		20
VIP	M=蒲地蓝 AND (M=小儿 OR 儿童 OR 未成年) AND (M=流行性腮腺炎 OR 痄腮)		14
Sinomed	蒲地蓝 AND (流行性腮腺炎 OR 痄腮) AND (小儿 OR 儿童)		23
Yiigle	主题=流行性腮腺炎 OR 痄腮) AND 主题=(蒲地蓝) AND 主题=(小儿 OR 儿童 OR 未成年)		0
Pubmed	(mumps[MeSH Terms]) OR (mumps[Title/Abstract]) OR (mumps virus[Title/Abstract]) OR (epidemic parotitis[Title/Abstract]) OR (epidemic parotitis virus[Title/Abstract])	#1	10403
	(Pudilan Xiaoyan Oral liquid[Title/Abstract]) OR (Pudilan[Title/Abstract])	#2	28
	(Children[Title/Abstract]) OR (kids[Title/Abstract]) OR (Juveniles[Title/Abstract])	#3	1200880
	#1 AND #2 AND #3	#4	1
Cochorane Library	MeSH descriptor: [Mumps] explode all trees	#1	102
	(mumps):ti,ab,kw OR (mumps virus):ti,ab,kw OR (epidemic parotitis):ti,ab,kw OR (epidemic parotitis virus):ti,ab,kw	#2	617
	#1 AND #2	#3	617
	(children):ti,ab,kw OR (kids):ti,ab,kw OR (juveniles):ti,ab,kw	#4	163100
	(Pudilan Xiaoyan Oral liquid):ti,ab,kw OR (Pudilan):ti,ab,kw	#5	10
	#3 AND #4 AND #5	#6	0
Embase	mumps/exp	#1	19672
	mumps:ti	#2	5245
	mumps virus:ti	#3	1976
	epidemic parotitis:ti	#4	356
	epidemic parotitis virus:ti	#5	46
	#1 OR #2 OR #3 OR #4 #5	#6	19737
	children	#7	2210207
	kids	#8	22329
	juveniles	#9	15019
	#7 OR #8 OR #9	#10	2232511
	Pudilan Xiaoyan Oral liquid	#11	25
	Pudilan	#12	44
	#11 OR #12	#13	44
	#6 AND #10 AND #13	#14	0
Web of Science	(((TS=(mumps)) OR TS=(mumps virus)) OR TS=(epidemic parotitis)) OR TS=(epidemic parotitis virus)	#1	3264
	(TS=(Pudilan Xiaoyan Oral liquid)) OR TS=(Pudilan)	#2	18
	((TS=(kids)) OR TS=(children)) OR TS=(juvenils)	#3	828033
	#1 AND #2 AND #3	#4	1
Google Scholar	(mumps OR mumps virus OR epidemic parotitis OR epidemic parotitis virus) AND (children OR kids OR juveniles) AND (Pudilan OR Pudilan Xiaoyan oral liquid))		2

Table S2 Information of components of PDL

MolID	MolName	OB(%)	DL	Herb
MOL005229	artemetin	49.55	0.48	Mongolian Dandelion Herb
MOL000006	luteolin	36.16	0.25	Mongolian Dandelion Herb
MOL000527	luteolin-4'-O-β-D-glucoside	35.94	0.79	Mongolian Dandelion Herb
MOL000098	quercetin	46.43	0.28	Mongolian Dandelion Herb
MOL002881	diosmetin	31.14	0.27	Mongolian Dandelion Herb
MOL005573	genkwanin	37.13	0.24	Mongolian Dandelion Herb
MOL012976	COUMESTROL	32.49	0.34	Mongolian Dandelion Herb
MOL008135	3，4-di-O-caffeoylquinic acid	49.62	0.69	Mongolian Dandelion Herb
MOL006554	taraxerol	38.40	0.77	Mongolian Dandelion Herb
MOL000449	Stigmasterol	43.83	0.76	Mongolian Dandelion Herb, Indigowoad Root, Baical skullcap root
MOL000359	sitosterol	36.91	0.75	Mongolian Dandelion Herb, Indigowoad Root, Baical skullcap root
MOL001986	β-sitosterol	33.94	0.70	Mongolian Dandelion Herb
NA1	isoetin	high	5*yes	Mongolian Dandelion Herb
NA2	3，4-dihydroxybenzoicacid	high	3*yes	Mongolian Dandelion Herb
NA3	methyl p-hydroxyphenylacetate	high	4*yes	Mongolian Dandelion Herb
NA4	ethyl p-hydroxyphenylacetate	high	4*yes	Mongolian Dandelion Herb
NA5	11β，13-dihydrotaraxinic acid	high	4*yes	Mongolian Dandelion Herb
NA6	artecalin	high	5*yes	Mongolian Dandelion Herb
NA7	arsanin	high	5*yes	Mongolian Dandelion Herb
NA8	taraxacin	high	5*yes	Mongolian Dandelion Herb
NA9	chloranthölide C	high	5*yes	Mongolian Dandelion Herb
MOL001458	coptisine	30.67	0.86	Corydalis bungeana, Baical skullcap root
MOL001463	Dihydrosanguinarine	59.31	0.86	Corydalis bungeana
MOL000217	(S)-Scoulerine	32.28	0.54	Corydalis bungeana
MOL004230	stylopine	48.25	0.85	Corydalis bungeana
MOL004763	Izoteolin	39.53	0.51	Corydalis bungeana
MOL000729	Oxysanguinarine	46.97	0.87	Corydalis bungeana
MOL000787	Fumarine	59.26	0.83	Corydalis bungeana
MOL000791	bicuculline	69.67	0.88	Corydalis bungeana
MOL008632	bungeanine	37.03	0.82	Corydalis bungeana
MOL008633	(S)-Cheilanthifoline	46.68	0.72	Corydalis bungeana
MOL008634	corycavine	67.18	0.84	Corydalis bungeana
MOL008635	coryincine	38.27	0.81	Corydalis bungeana
MOL008636	corynoline	30.53	0.85	Corydalis bungeana
MOL008637	d-isocorynoline	36.38	0.86	Corydalis bungeana
MOL008638	13-epicorynoline	34.04	0.85	Corydalis bungeana

MOL008640	isocorynoline	47.44	0.86	Corydalis bungeana
MOL008641	Norsanguinarine	45.04	0.86	Corydalis bungeana
MOL008642	noryuziphine	51.93	0.2	Corydalis bungeana
MOL008645	yuziphine	84.32	0.23	Corydalis bungeana
MOL008646	6-acetonyldihydrosanguinarine	50.35	0.83	Corydalis bungeana
MOL008647	Moupinamide	86.71	0.26	Corydalis bungeana
MOL008648	acetylcorynoline	43.72	0.83	Corydalis bungeana
MOL001689	acacetin	34.97	0.24	Indigowoad Root, Baical skullcap root
MOL002322	isovitexin	31.29	0.72	Indigowoad Root
MOL001721	Isaindigodione	60.12	0.41	Indigowoad Root
MOL001722	2-O-beta-D-glucopyranosyl-2H-1,4-benzoxazin-3(4H)-one	43.62	0.31	Indigowoad Root
MOL001726	pinoresinol-4-O-beta-D-apiosyl-beta-D-glucopyranoside	36.45	0.51	Indigowoad Root
MOL001728	3-[2′ -(5′ - hydroxymethyl) furyl] -1 (2H) -isoquinolinone-7-O-BETA-D-glucoside_qt	51.74	0.18	Indigowoad Root
MOL001733	EUPATORIN	30.23	0.37	Indigowoad Root
MOL001734	3-[[(2R,3R,5R,6S)-3,5-dihydroxy-6-(1H-indol-3-yloxy)-4-oxooxan-2-yl]methoxy]-3-oxopropanoic acid	85.87	0.47	Indigowoad Root
MOL001735	Dinatin	30.97	0.27	Indigowoad Root
MOL001736	(-)-taxifolin	60.51	0.27	Indigowoad Root
MOL001749	ZINC03860434	43.59	0.35	Indigowoad Root
MOL001750	glucobrassicin	66.02	0.48	Indigowoad Root
MOL001755	24-Ethylcholest-4-en-3-one	36.08	0.76	Indigowoad Root
MOL001756	quindoline	33.17	0.22	Indigowoad Root
MOL001767	hydroxyindirubin	63.37	0.3	Indigowoad Root
MOL001769	beta-sitosterol dodecantate	34.57	0.57	Indigowoad Root
MOL001771	poriferast-5-en-3beta-ol	36.91	0.75	Indigowoad Root
MOL001774	Ineketone	37.14	0.3	Indigowoad Root
MOL001779	Sinoacutine	49.11	0.46	Indigowoad Root
MOL001781	Indigo	38.2	0.26	Indigowoad Root
MOL001782	(2Z)-2-(2-oxoindolin-3-ylidene)indolin-3-one	48.4	0.26	Indigowoad Root
MOL001783	2-(9-((3-methyl-2-oxopent-3-en-1-yl)oxy)-2-oxo-1,2,8,9-tetrahydrofuro[2,3-h]quinolin-8-yl)propan-2-yl acetate	64	0.57	Indigowoad Root
MOL001790	Linarin	39.84	0.71	Indigowoad Root
MOL001792	DFV	32.76	0.18	Indigowoad Root
MOL001793	(E)-2-[(3-indole)cyanomethylene-]-3-indolinone	54.59	0.32	Indigowoad Root
MOL001798	neohesperidin_qt	71.17	0.27	Indigowoad Root
MOL001800	rosasterol	35.87	0.75	Indigowoad Root
MOL001803	Sinensetin	50.56	0.45	Indigowoad Root
MOL001804	Stigmasta-5,22-diene-3beta,7alpha-diol	43.04	0.82	Indigowoad Root
MOL001806	Stigmasta-5,22-diene-3beta,7beta-diol	42.56	0.83	Indigowoad Root

MOL001810	6-(3-oxoindolin-2-ylidene)indolo[2,1-b]quinazolin-12-one	45.28	0.89	Indigowoad Root
MOL001814	(E)-3-(3,5-dimethoxy-4-hydroxy-benzylidene)-2-indolinone	57.18	0.25	Indigowoad Root
MOL001820	(E)-3-(3,5-dimethoxy-4-hydroxyb-enzylidene)-2-indolinone	65.17	0.25	Indigowoad Root
MOL001828	3-[(3,5-dimethoxy-4-oxo-1-cyclohexa-2,5-dienylidene)methyl]-2,4-dihydro-1H-pyrrolo[2,1-b]quinazolin-9-one	51.84	0.56	Indigowoad Root
MOL001833	Glucobrassicin-1-Sulfonate_qt	42.52	0.24	Indigowoad Root
MOL000358	beta-sitosterol	36.91	0.75	Indigowoad Root, Baical skullcap root
MOL000953	CLR	37.87	0.68	Indigowoad Root
MOL000173	wogonin	30.68	0.23	Baical skullcap root
MOL000228	(2R)-7-hydroxy-5-methoxy-2-phenylchroman-4-one	55.23	0.2	Baical skullcap root
MOL002714	baicalein	33.52	0.21	Baical skullcap root
MOL002908	5,8,2'-Trihydroxy-7-methoxyflavone	37.01	0.27	Baical skullcap root
MOL002909	5,7,2,5-tetrahydroxy-8,6-dimethoxyflavone	33.82	0.45	Baical skullcap root
MOL002910	Carthamidin	41.15	0.24	Baical skullcap root
MOL002911	2,6,2',4'-tetrahydroxy-6'-methoxychaleone	69.04	0.22	Baical skullcap root
MOL002913	Dihydrobaicalin_qt	40.04	0.21	Baical skullcap root
MOL002914	Eriodyctiol (flavanone)	41.35	0.24	Baical skullcap root
MOL002915	Salvigenin	49.07	0.33	Baical skullcap root
MOL002917	5,2',6'-Trihydroxy-7,8-dimethoxyflavone	45.05	0.33	Baical skullcap root
MOL002925	5,7,2',6'-Tetrahydroxyflavone	37.01	0.24	Baical skullcap root
MOL002926	dihydrooroxylin A	38.72	0.23	Baical skullcap root
MOL002927	Skullcapflavone II	69.51	0.44	Baical skullcap root
MOL002928	oroxylin a	41.37	0.23	Baical skullcap root
MOL002932	Panicolin	76.26	0.29	Baical skullcap root
MOL002933	5,7,4'-Trihydroxy-8-methoxyflavone	36.56	0.27	Baical skullcap root
MOL002934	NEOBAICALEIN	104.34	0.44	Baical skullcap root
MOL002937	DIHYDROOROXYLIN	66.06	0.23	Baical skullcap root
MOL000525	Norwogonin	39.4	0.21	Baical skullcap root
MOL000552	5,2'-Dihydroxy-6,7,8-trimethoxyflavone	31.71	0.35	Baical skullcap root
MOL000073	ent-Epicatechin	48.96	0.24	Baical skullcap root
MOL001490	bis[(2S)-2-ethylhexyl] benzene-1,2-dicarboxylate	43.59	0.35	Baical skullcap root
MOL001506	Supraene	33.55	0.42	Baical skullcap root
MOL002879	Diop	43.59	0.39	Baical skullcap root
MOL002897	epiberberine	43.09	0.78	Baical skullcap root
MOL008206	Moslosooflavone	44.09	0.25	Baical skullcap root
MOL010415	11,13-Eicosadienoic acid, methyl ester	39.28	0.23	Baical skullcap root
MOL012245	5,7,4'-trihydroxy-6-methoxyflavanone	36.63	0.27	Baical skullcap root
MOL012246	5,7,4'-trihydroxy-8-methoxyflavanone	74.24	0.26	Baical skullcap root
MOL012266	rivularin	37.94	0.37	Baical skullcap root

Table S3 Active sites' coordinates and grid boxes' sizes of molecular docking

Targets	ALB	IL6	IL1B	VEGFA	ESR1	HSP90AA1	ERBB2	Mumps virus
PDBID<br>Coordinates of active sites / sizes of grid boxes	6ZL1	1IL6	1HIb	1BJ1	1L2I	7L7J	7MN6	7EWQ
center_x	0.398	-20.472	-20.472	23.953	-20.472	-20.472	-20.472	-13.417
center_y	15.597	-14.306	-14.306	-22.048	-14.306	-14.306	-7.861	-7.861
center_z	26.458	6.917	10.583	-4.360	6.278	6.278	15.194	15.194
size_x	116	96	98	52	62	96	64	78
size_y	126	58	58	60	52	98	124	58
size_z	76	66	78	74	70	126	86	80

Table S4 Binding energy of molecular docking

Targets		ALB	IL6	IL1B	VEGFA	ESR1	HSP90AA1	ERBB2	Mumps virus
Ingredients/drugs	PDBID	6ZL1	1IL6	1HIb	1BJ1	1L2I	7L7J	7MN6	7EWQ
MOL000098	quercetin	-8.1	-6.9	-7.1	-7.3	-7.8	-9.9	-7.9	-8.6
NA1	isoetin	-8.3	-7.0	-7.3	-7.1	-8.5	-9.7	-8.0	-8.3
NA8	taraxacin	-8.1	-6.4	-7.0	-6.8	-9.2	-9.2	-7.7	-7.4
NA4	ethyl p-hydroxyphenylacetate	-6.2	-5.0	-5.2	-4.9	-5.9	-6.6	-5.8	-5.4
NA5	11β，13-dihydrotaraxinic acid	-7.7	-6.2	-7.0	-7.0	-7.4	-8.6	-7.5	-7.6
NA9	chloranthotide C	-8.0	-6.6	-6.3	-6.8	-8.4	-8.9	-7.2	-7.7
NA6	artecalin	-8.3	-6.6	-6.2	-6.8	-8.2	-8.3	-7.0	-7.1
MOL000006	luteolin	-8.4	-6.8	-7.2	-7.1	-8.5	-9.5	-7.9	-8.1
NA3	methyl p-hydroxyphenylacetate	-5.9	-4.9	-5.2	-4.7	-5.9	-6.3	-5.6	-5.5
MOL000173	wogonin	-8.0	-6.2	-6.3	-6.9	-8.0	-8.7	-7.8	-7.2
NA7	arsanin	-8.2	-6.6	-6.3	-6.8	-8.2	-8.3	-8.0	-7.5
ribavirin	ribavirin	-6.6	-6.0	-6.4	-6.2	-6.6	-8.1	-7.3	-6.8
ganciclovir	ganciclovir	-6.1	-5.5	-5.9	-6.0	-6.3	-7.2	-6.6	-6.7

Section and Topic	Item #	Checklist item	Location where item is reported
**TITLE**			
Title	1	Identify the report as a systematic review.	Page 1
**ABSTRACT**			
Abstract	2	See the PRISMA 2020 for Abstracts checklist.	Page 1, 2
**INTRODUCTION**			
Rationale	3	Describe the rationale for the review in the context of existing knowledge.	Page 2, 3
Objectives	4	Provide an explicit statement of the objective(s) or question(s) the review addresses.	Page 3
**METHODS**			
Eligibility criteria	5	Specify the inclusion and exclusion criteria for the review and how studies were grouped for the syntheses.	Page 4
Information sources	6	Specify all databases, registers, websites, organisations, reference lists and other sources searched or consulted to identify studies. Specify the date when each source was last searched or consulted.	Page 4
Search strategy	7	Present the full search strategies for all databases, registers and websites, including any filters and limits used.	Page 4
Selection process	8	Specify the methods used to decide whether a study met the inclusion criteria of the review, including how many reviewers screened each record and each report retrieved, whether they worked independently, and if applicable, details of automation tools used in the process.	Page 4, 5
Data collection process	9	Specify the methods used to collect data from reports, including how many reviewers collected data from each report, whether they worked independently, any processes for obtaining or confirming data from study investigators, and if applicable, details of automation tools used in the process.	Page 4, 5
Data items	10a	List and define all outcomes for which data were sought. Specify whether all results that were compatible with each outcome domain in each study were sought (e.g. for all measures, time points, analyses), and if not, the methods used to decide which results to collect.	Page 4, 5
	10b	List and define all other variables for which data were sought (e.g. participant and intervention characteristics, funding sources). Describe any assumptions made about any missing or unclear	Page 4, 5

		information.	
Study risk of bias assessment	11	Specify the methods used to assess risk of bias in the included studies, including details of the tool(s) used, how many reviewers assessed each study and whether they worked independently, and if applicable, details of automation tools used in the process.	Page 5
Effect measures	12	Specify for each outcome the effect measure(s) (e.g. risk ratio, mean difference) used in the synthesis or presentation of results.	Page 5
Synthesis methods	13a	Describe the processes used to decide which studies were eligible for each synthesis (e.g. tabulating the study intervention characteristics and comparing against the planned groups for each synthesis (item #5)).	Page 5
	13b	Describe any methods required to prepare the data for presentation or synthesis, such as handling of missing summary statistics, or data conversions.	Page 5
	13c	Describe any methods used to tabulate or visually display results of individual studies and syntheses.	Page 5
	13d	Describe any methods used to synthesize results and provide a rationale for the choice(s). If meta-analysis was performed, describe the model(s), method(s) to identify the presence and extent of statistical heterogeneity, and software package(s) used.	Page 4
	13e	Describe any methods used to explore possible causes of heterogeneity among study results (e.g. subgroup analysis, meta-regression).	Page 5
	13f	Describe any sensitivity analyses conducted to assess robustness of the synthesized results.	Page 5
Reporting bias assessment	14	Describe any methods used to assess risk of bias due to missing results in a synthesis (arising from reporting biases).	Page 5
Certainty assessment	15	Describe any methods used to assess certainty (or confidence) in the body of evidence for an outcome.	Page 5
**RESULTS**			
Study selection	16a	Describe the results of the search and selection process, from the number of records identified in the search to the number of studies included in the review, ideally using a flow diagram.	Page 6, 7
	16b	Cite studies that might appear to meet the inclusion criteria, but which were excluded, and explain why they were excluded.	Page 7
Study characteristics	17	Cite each included study and present its characteristics.	Page 7, 8
Risk of bias in	18	Present assessments of risk of bias for each included study.	Page 9

studies			
Results of individual studies	19	For all outcomes, present, for each study: (a) summary statistics for each group (where appropriate) and (b) an effect estimate and its precision (e.g. confidence/credible interval), ideally using structured tables or plots.	Page 9~13
Results of syntheses	20a	For each synthesis, briefly summarise the characteristics and risk of bias among contributing studies.	Page 9~13
	20b	Present results of all statistical syntheses conducted. If meta-analysis was done, present for each the summary estimate and its precision (e.g. confidence/credible interval) and measures of statistical heterogeneity. If comparing groups, describe the direction of the effect.	Page 9~13
	20c	Present results of all investigations of possible causes of heterogeneity among study results.	Page 9~ 13
	20d	Present results of all sensitivity analyses conducted to assess the robustness of the synthesized results.	Page 14
Reporting biases	21	Present assessments of risk of bias due to missing results (arising from reporting biases) for each synthesis assessed.	Page 13, 14
Certainty of evidence	22	Present assessments of certainty (or confidence) in the body of evidence for each outcome assessed.	Page14
**DISCUSSION**			
Discussion	23a	Provide a general interpretation of the results in the context of other evidence.	Page 19
	23b	Discuss any limitations of the evidence included in the review.	Page 19~20
	23c	Discuss any limitations of the review processes used.	Page 19~20
	23d	Discuss implications of the results for practice, policy, and future research.	Page 20
**OTHER INFORMATION**			
Registration and protocol	24a	Provide registration information for the review, including register name and registration number, or state that the review was not registered.	Page 4
	24b	Indicate where the review protocol can be accessed, or state that a protocol was not prepared.	Page 4
	24c	Describe and explain any amendments to information provided at registration or in the protocol.	Page 4
Support	25	Describe sources of financial or non-financial support for the review, and the role of the funders or sponsors in the review.	Page 21
Competing interests	26	Declare any competing interests of review authors.	Page 26

Availability of data, code and other materials	27	Report which of the following are publicly available and where they can be found: template data collection forms; data extracted from included studies; data used for all analyses; analytic code; any other materials used in the review.	Page 22

From: Page MJ, McKenzie JE, Bossuyt PM, Boutron I, Hoffmann TC, Mulrow CD, et al. The PRISMA 2020 statement: an updated guideline for reporting systematic reviews. BMJ 2021;372:n71. doi: 10.1136/bmj.n71

For more information, visit: http://www.prisma-statement.org/
